# A Multi-Scale Cross-Band Defense System Integrating Decoupled Visible, Dynamic Infrared Camouflage and Electromagnetic Shielding

**DOI:** 10.1007/s40820-025-01961-4

**Published:** 2026-01-13

**Authors:** Junlin Liu, Shujuan Tan, Xinrui Yang, Jiajie Zhu, Xin Yan, Tianyu Chen, Guangbin Ji

**Affiliations:** College of Material Science and Technology, Nanjing University of Aeronautics and Astronautics, Nanjing, 210016 People’s Republic of China

**Keywords:** Visible camouflage, Adaptive IR camouflage, Cu_2_O/CNT composite film, Electrothermal energy conversion, EMI shielding

## Abstract

Cross-band camouflage technology is a critical necessity, enabling personnel and equipment to evade detection across evolving surveillance systems, thereby enhancing survivability and mission success. Herein, this work develops a layer-structured composite system based on carbon nanotube (CNT) film comprising ionic liquid (IL) interlayer for infrared (IR) modulation and surface-engineered Cu_2_O nanoparticles for visible camouflage. The CNT/IL/CNT architecture enables reversible IR emissivity switching (Δε≈0.55) through electrically driven ion intercalation/deintercalation within 2 s, while spray-coated Cu_2_O nanoparticles (100 ~ 400 nm diameter) on the top CNT film layer generate rich structure colors with 90% IR transmittance. This spectral-decoupling design overcomes the traditional trade-off between color visibility and IR transmittance observed in pigment-based systems. Remarkably, due to physical interface coupling, the Cu_2_O-coated layer-structured system maintains exceptional electrical conductivity, enabling simultaneous electromagnetic interference shielding and electrothermal energy conversion. The integrated system demonstrates long-term operational stability. By unifying visible-IR camouflage, electromagnetic protection, and energy management in a lightweight platform, this work provides an important paradigm for cross-band camouflage technologies.

**Supplementary Information:**

The online version contains supplementary material available at 10.1007/s40820-025-01961-4.

## Highlights

- A multi-scale cross-band military lightweight camouflage system is constructed via elaborate hierarchical structure design.
- This system can achieve rich color presentation to meet the visible camouflage requirements in different backgrounds.
- This system can simultaneously achieve an infrared emissivity modulation of Δε > 0.5, achieving excellent dynamic infrared stealth.
- This system characterized by its light weight can also achieve efficient electromagnetic interference shielding and excellent electrothermal conversion.

**Supplementary Information:**

The online version contains supplementary material available at 10.1007/s40820-025-01961-4.

## Graphical abstract

**Figure:**
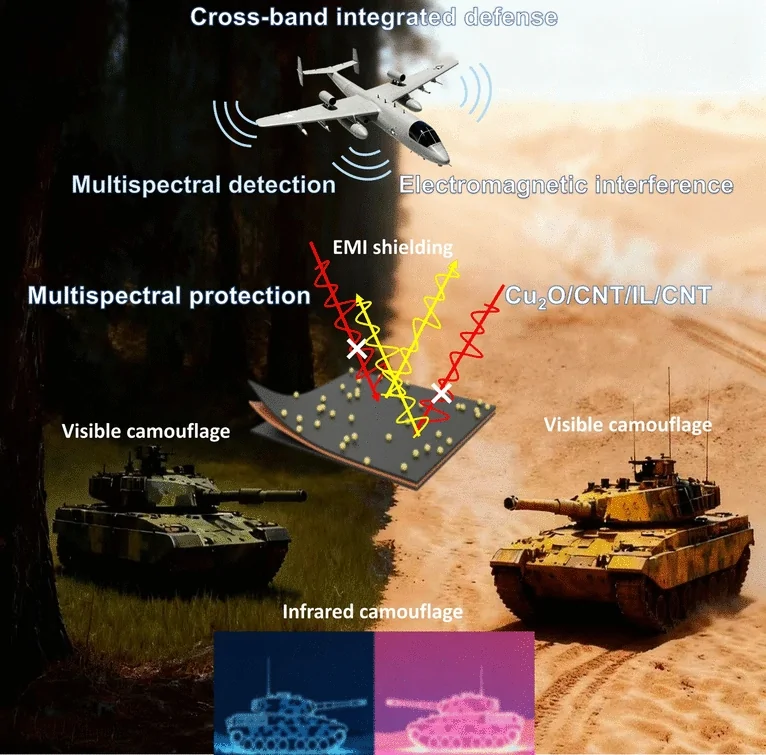

## Introduction

Modern warfare increasingly depends on integrated multi-spectral detection systems that simultaneously exploit visible, infrared (IR), and microwave detection bands, rendering conventional single-band camouflage strategies obsolete [1, 2]. This trend imposes critical demands for both chromatic diversity in the visible spectrum and dynamic tunability in the IR spectrum to avoid detection [3–6]. Effective electromagnetic interference (EMI) shielding is also required to prevent information leakage and preserve electronic integrity in signal-saturated combat environments [7–10]. In addition, under extreme conditions, efficient electrothermal conversion enables essential battlefield capabilities including randomized thermal signatures and ice removal, which are frequently overlooked in purely optical engineering systems [11].

Traditional metallic coatings, while offering low IR emissivity, suffer from intrinsic limitations. Their high specular reflectance compromises visible concealment, while static IR optical properties preclude real-time adaptability [12]. Conversely, electrochromic polymers optimized for visible camouflage exhibit fundamentally constrained IR modulation depth due to inherent molecular absorption characteristics [13]. Meanwhile, traditional hybridization methodologies based on above material systems for EMI shielding performance, such as metallic nanowire integration, impose prohibitive areal densities and severely compromise mechanical flexibility [14–16]. This technological difficulty highlights an urgent need for advanced materials capable of independent spectral regulation across electromagnetic domains that is essential not only for next-generation military camouflage but also for aerospace thermal management and intelligent wearable systems [17–19].

Carbon-based materials become the preferred materials for intelligent IR camouflage due to the unique electrochemical activity and tunable carrier absorption characteristics [20, 21]. The pioneering work of Coskun Kocabas et al. indicated that the intercalation and deintercalation of ionic liquids (IL) into multilayer graphene films can achieve reversible IR emissivity changes (Δε = 0.45 ~ 0.50) through the regulation of intraband transition [22]. Subsequently, Sun. et al. utilized the controllable charge transport of CNT films to achieve a response time of less than 2 s in IR emissivity modulation, which is superior to traditional electrochromic polymers [23]. Moreover, their high electrical conductivity is crucial to EMI shielding. However, due to the strong absorption of free carriers, CNT films retain more than 90% of the visible absorption characteristics, resulting in the visual black characteristics and limitations in the field of visible camouflage [24]. Thus, it is particularly important to color carbon-based materials. Conventional chemical coloration approaches, such as coating CNT films with organic dyes or inorganic pigments, necessitate preliminary deposition of a reflective white underlayer (TiO_2_ or BaSO_4_) to counteract the intrinsic optical absorption [25, 26]. This process invariably introduces microscale thickness (about 10 μm) and induces significant IR transmission hindrance. Alternative non-pigment coloration strategies, including graphene plasmonic resonators and Fabry–Perot cavities, partially mitigate pigment-derived limitations yet introduce new compromises. Multi-layer Fabry–Perot configurations demand stringent thickness control (± 5 nm tolerance) to generate target hues, while their essential dielectric spacers suppress IR transmittance to approximately 70% [27–29]. Even recently reported SiO_2_ photonic crystals exhibit evident IR transmission degradation when nanoparticles stacking densities required for angle-insensitive coloration on CNTs substrates are implemented [30]. Above-mentioned central challenge stems from the inherent conflict between achieving sufficient coloration thickness for visible coloration and maintaining ultra-thin coverage for IR transmittance [31], severely limiting the practical implementation of existing carbon-based systems in military camouflage applications.

**Fig. 1:**
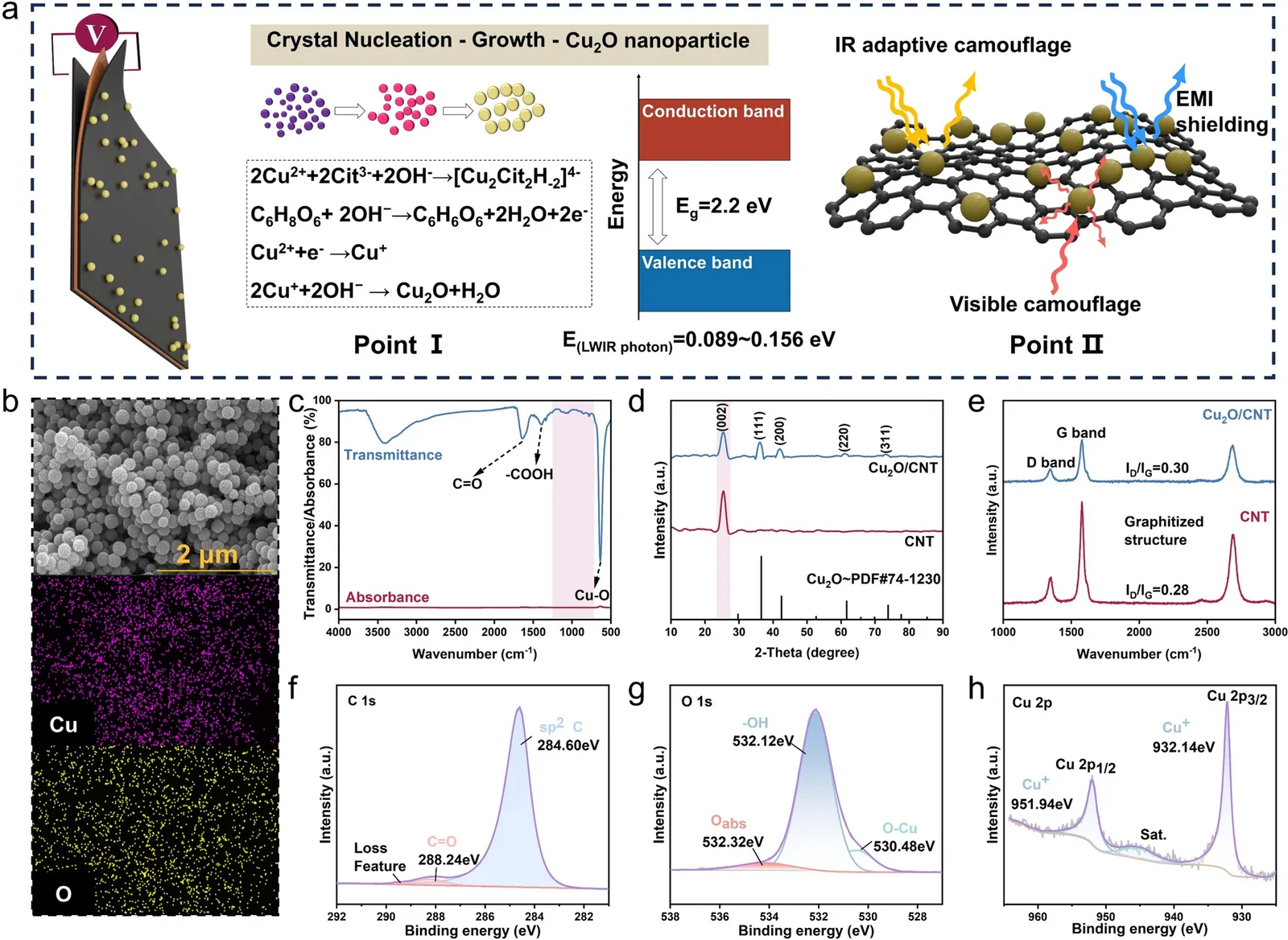
The design concept of multi-scale cross-band defense system and microscopic characterization. **a** Material system and design concept. **b** SEM image and EDS mapping of Cu_2_O nanoparticles. **c** FTIR spectrum of Cu_2_O nanoparticles. **d** Comparison of XRD patterns between Cu_2_O/CNT composite film and CNT film. **e** Comparison of Raman spectrum between Cu_2_O/CNT composite film and CNT film. XPS spectrum of Cu_2_O/CNT composite film **f** C 1* s* fine spectrum. **g** O 1* s* fine spectrum. **h** Cu 2*p* fine spectrum

To address these interconnected challenges, this work spatially decouples IR modulation and visible coloration functions. Considering that the coloring layer would impede IR transmission, Cu_2_O nanoparticles with a high refractive index are chosen, expecting to achieve rich colors through non-close-packed few-layer arrangement. Therefore, we develop a layered architecture integrating a CNT/IL/CNT sandwich configuration with surface-engineered Cu_2_O nano-coatings (Point l, Fig. 1a). The parallel CNT films with IL interlayers enable excellent dynamic IR emissivity modulation (Δε > 0.50 at 8 ~ 14 μm, LWIR) via reversible ion intercalation, while spray-deposited Cu_2_O nanoparticles layer generates rich angle-insensitive structure colors through diameter-dependent Mie scattering, sustaining exceeding 90% transmittance in LWIR band. Such a structure design obviates the need for conventional reflective substrate during coloration process. Critically, the preserved internal conductive network of CNTs in composite system enables simultaneous realization of broadband EMI shielding (> 70 dB in the X-band) and efficient electrothermal conversion (Point ll Fig. 1a). By integrating these functionalities into a lightweight system, our work transcends conventional material design paradigms to provide a versatile solution for advanced multi-spectral camouflage.

## Experimental Section

### Materials

All chemical reagents are used as received at the analytical purity level, and no further purification treatment is necessary. Copper (II) acetate monohydrate (Cu(CH_3_COO)_2_·H_2_O, ≥ 99.0%), trisodium citrate dihydrate (C_6_H_5_Na_3_O_7_·2H_2_O, ≥ 99.0%), polyvinylpyrrolidone (PVP, MW ~ 55,000/58,000), sodium hydroxide (NaOH, ≥ 96.0%), potassium hydroxide (KOH, ≥ 96.0%), and L-ascorbic acid (≥ 99.0%) are purchased from Aladdin Biochemical Technology Co., Ltd. Thiophene (C_4_H_4_S, ≥ 99.0%) and ferrocene (C_10_H_10_Fe, ≥ 98.0%) are sourced from Merck Group. Anhydrous ethanol (≥ 99.7%), methanol (≥ 99.5%), ethylene glycol (≥ 99.0%), polyurethane (PU, solid content: 55%), polyvinyl alcohol (PVA, ≥ 99%), isopropanol (≥ 99.7%), deionized water, polydimethylsiloxane (PDMS, ≥ 99%), and ionic liquid [EMIM] [TFSI] (≥ 99.8%) are procured from Aladdin. Wet-process polyethylene (PE) separator (20 μm thickness), copper foil conductive tape (electrolytic grade), flat coating brushes, and pneumatic airbrushes (0.3 mm nozzle) are supplied by Nanjing Lattice Materials Technology Co., Ltd.

### Fabrication and Functionalization of CNT Films

Carbon nanotube (CNT) films are synthesized via floating catalyst chemical vapor deposition (FCCVD). A precursor solution containing ethanol (carbon source), thiophene (growth promoter), and ferrocene (catalyst precursor) is injected at 5 mL min^−1^ into a tube furnace (OTF-1600X, MTI Corp., China) maintained at 1200 °C under H_2_/Ar atmosphere using a peristaltic pump (BT100-2J, Longer Precision Pump Co., China). Resultant continuous fluffy CNT assemblies are collected on a rotating drum (KSL-1100X-S, MTI Corp., China) and densified through liquid-phase compaction in methanol/water solution. Residual impurities are removed by immersion in 1.5 M KOH (0.5 h, 20 °C) followed by thorough deionized water rinsing until neutral effluent. Controlled surface functionalization is achieved via mixed-acid (H_2_SO_4_:HNO_3_, 2:1 v/v) treatment. CNT films are immersed in 100 mL solutions containing 15, 30, 60, and 90 mL mixed acid diluted with deionized water under magnetic stirring (RCT basic, IKA, Germany; 48 h, room temperature). After functionalization, CNT films are rinsed to neutral pH with deionized water and vacuum-dried (DZF-6050, Jinghong Instruments, China; 60 °C, 12 h). Samples are designated MACNT-1, MACNT-2, MACNT-3, and MACNT-4 with increasing mixed-acid volume.

### Synthesis of Cu_2_O Nanoparticles

In a 500 mL beaker, 0.7 g Cu(CH_3_COO)_2_·H_2_O, 1.0 g C_6_H_5_Na_3_O_7_·2H_2_O, and 3.0 g PVP (Mw = 58,000) are sequentially dissolved in ethylene glycol/water mixed solvent (mass ratio 3:10) under magnetic stirring (RCT basic, IKA, Germany; 300 rpm) until homogeneous solution forms. A 0.5 mol L^−1^ NaOH solution (40 mL) is added dropwise over 6 min via a peristaltic pump (BT100-2J, Longer Precision Pump Co., China), followed by agitation at 600 rpm for 10 min. After restoring stirring to 300 rpm, 15 mL of 0.12 mol L^−1^ L-ascorbic acid solution is introduced at constant rate over 5 min. An equal volume of L-ascorbic acid solution is poured 5 min later, and reaction proceeds for 2 h. The product is centrifuged (Centrifuge 5424R, Eppendorf, Germany; 8,000 rpm, 15 min), washed thrice with ethanol/water mixture (1:1 mass ratio; 8000 rpm, 5 min), and vacuum-dried (DZF-6050, Jinghong Instruments, China; 60 °C, 3 h) to yield orange-brown powder. Cu_2_O nanoparticles with varying morphologies/diameters are prepared by controlling Cu(CH_3_COO)_2_·H₂O to C_6_H_5_Na_3_O_7_·2H_2_O ratio, PVP molecular weight, and L-ascorbic acid dropping rate.

### Fabrication of Cu_2_O/CNT Composite Films

Cu_2_O nanoparticles (0.1 g) are dispersed in anhydrous ethanol (4.9 g) under mechanical stirring (RCT basic, IKA, Germany; 300 rpm) and ultrasonication (KQ-500DE, Kunshan Ultrasonic Instruments, China; 15 min). The dispersion is pneumatically sprayed onto CNT films using a 0.3 mm nozzle airbrush (0.3 MPa pressure, 40 cm spraying distance) with fifteen sequential passes. Subsequent drying at 60 °C for 3 min produces Cu_2_O/CNT composite films. Fabricated purple, blue, green, yellow and orange composite films are designated CC-1, CC-2, CC-3, CC-4, and CC-5, respectively.

### Assembly of IR Electrochromic Devices

IR electrochromic devices are assembled in sandwich configuration, comprising Cu_2_O/CNT composite film as modulation working electrode and pristine CNT film (polished via dust-free paper dry treatment) as ion storage counter electrode. The IL electrolyte [EMIM] [TFSI] is uniformly coated on wet-process PE separator (20 μm thickness) with flat coating brush. Electrodes are laminated onto both sides of electrolyte-coated separator by capillary force. Electrical leads connected via copper foil conductive tape are interfaced with DC power supply (DP832, Rigol Technologies, China) to apply −3 to + 3 V bias voltages (30 s holding time).

### Characterizations

Digital photographs are captured using a Huawei smartphone. UV–Vis-NIR diffuse reflectance spectroscopy (Shimadzu UV-3600, Japan) measures reflectance across 380 ~ 2500 nm, with chromaticity coordinates plotted via the CIE 1931 standard. Surface morphology is characterized by focused ion beam-scanning electron microscopy (Thermo Fisher Scientific Helios G4, USA) coupled with energy-dispersive X-ray spectroscopy (EDS, Oxford Instruments, UK) for elemental mapping. Crystal structures are analyzed by X-ray diffraction (Rigaku Ultima IV, Japan; Cu K*α* radiation, *λ* = 1.5406 Å, 10 °min^−1^ scan rate). Raman spectroscopy (Renishaw inVia, UK) further assesses defect density. Chemical states are probed via X-ray photoelectron spectroscopy (Thermo Fisher Scientific ESCALAB Xi + , USA), with binding energies calibrated against the C 1*s* peak (284.8 eV). Fourier-transform IR spectroscopy (FTIR, Bruker Vertex 70, Germany) evaluates IR absorbance/transmittance. Four-probe resistance meter (Keithley 2400, USA) is used to evaluate electrical conductivity. Mechanical properties are characterized using a universal testing machine (Instron 5969, USA). Actual temperature is monitored by thermocouples (OMEGA Engineering, USA). Hydrophobicity is quantified by contact angle measurements (Krüss DSA100, Germany).

## Results and Discussion

### Integrated Material System Design and Micro-Characterization Analysis

Cu_2_O possesses the potential to achieve abundant structure colors of textiles at low spraying density [32]. As a semiconductor material, the valence band top of Cu_2_O is mainly composed of Cu_3d_ state electrons and O_2p_ state electrons, while the conduction band bottom results from the hybridization of Cu_4p_ state electrons and O_2p_ state electrons [33]. Its band gap is 2.2 eV, while the LWIR photon energy is 0.089 ~ 0.156 eV. Thus, there is no obvious characteristic absorption of Cu_2_O in LWIR band (Point l**,** Fig. 1a). In order to prepare Cu_2_O nanoparticles with regular morphology and diameter to achieve structure coloration [34], this work modifies a typical two-step liquid-phase reduction method at room temperature [35]. The reaction system exhibits observable color transitions during liquid-phase synthesis process (Fig. S1a–g). Final powder product (0.1 g) dispersed in ethanol (30 mL) can form a stable colloid (3.33 mg mL^−1^), displaying pronounced Tyndall effect under laser irradiation (Fig. S1h). This scattering phenomenon originates from monodisperse citrate-modified Cu_2_O nanoparticles in ethanol, where the diameter-laser wavelength relationship and high refractive index contrast between Cu_2_O (*n* = 2.7) and ethanol (*n* = 1.36) synergistically enhance scattering cross sections [36].

Synthesis using 0.7 g copper (ll) acetate monohydrate and 1 g trisodium citrate dihydrate yields 0.2425 g of orange-brown nanoparticles, representing a 96% yield relative to theoretical prediction (Fig. S1i). Scanning electron microscopy (SEM) reveals monodisperse nanospheres, while energy-dispersive X-ray spectroscopy (EDS) elemental mapping confirms exclusive presence of Cu and O elements without impurities (Fig. 1b). Reflectance spectra (380 ~ 780 nm, Fig. S1i) demonstrates wavelength-dependent behavior. Low reflectance at shorter wavelength progressively increases toward longer wavelength, consistent with predominant red-light reflection causing orange-brown visual appearance. To investigate the spectral characteristic, Cu_2_O is characterized by Fourier-transform infrared spectroscopy (Fig. 1c). The peak at 630 cm^−1^ corresponds to the telescopic vibration of Cu–O bond in Cu_2_O. Peaks at 1400 and 1636 cm^−1^, respectively, correspond to asymmetric stretching vibration with carboxyl in citrate and absorption of carbonyl in PVP, which proves that synthesized Cu_2_O contains organic ligands from PVP and citrate. Furthermore, the spectral transmittance of Cu_2_O exceeds 90% in LWIR band, aligning with above-mentioned electronic band characteristics.

Figure S2 presents the preparation of CNT film [37] as well as the surface properties and spectral characteristics. Cu_2_O/CNT composite films are fabricated by air-spray deposition of Cu_2_O/ethanol dispersion onto CNT films (Fig. S3a). To confirm the successful construction of composite films, multi-dimensional characterizations are carried out. X-ray diffraction (XRD, Fig. 1d) identifies the characteristic (002) graphitic peak at 25.6° for both pristine CNT film and Cu_2_O/CNT composite film, confirming crystallinity preservation of CNT film. Composite film-specific diffraction peaks match cubic Cu_2_O (PDF#74–1230), with dominant (111) facet intensity verifying phase-pure deposition. Raman spectroscopy (Fig. 1e) indicates structure integrity retention, evidenced by consistent D-band (1350 cm⁻^1^) to G-band (1580 cm⁻^1^) intensity ratios (I_D_/I_G_ = 0.30 for composite film versus 0.28 for pristine CNT film), confirming minimal defect introduction during processing via physical decoupling. X-ray photoelectron spectroscopy (XPS) survey scan confirms the presence of C, O, and Cu (Figs. 1f-h and S3b). High-resolution C 1*s* spectra are deconvoluted into dominant *sp*^2^-hybridized carbon peak at 284.60 eV (graphitic CNT backbone) and minor C=O component at 288.24 eV (surface oxidation from CVD growth). Cu 2*p* fine spectra exhibits Cu 2*p*_3/2_ and 2*p*_1/2_ peaks at 932.14 and 951.94 eV, respectively. Negligible satellite peaks near 945 eV confirms Cu^+^ valence state. O 1*s* spectral decomposition identifies three components, including lattice oxygen at 530.48 eV (Cu–O bonding in Cu_2_O), chemisorbed oxygen at 532.32 eV (surface-adsorbed O_2_/H_2_O) and hydroxyl oxygen at 532.12 eV (interfacial OH⁻ from H₂O dissociation). The hydroxyl formation originates from interfacial charge transfer stabilizing dissociated water molecules at CNT/Cu_2_O junctions. These multi-scale characterizations conclusively validate successful Cu_2_O nanoparticles coating onto CNT films.

### Multicolor Presentation and Mechanism Investigation

Section 3.1 has confirmed the effectiveness of two-step liquid-phase reduction method in preparing Cu_2_O nanoparticles with regular appearance and diameter, and verified the feasibility of air-spray deposition method in the construction of composite films. To realize visible camouflage in multiple backgrounds, five kinds of Cu_2_O/CNT composite films with different colors are obtained by adjusting the parameters of the preparation process of Cu_2_O nanoparticles.

**Fig. 2:**
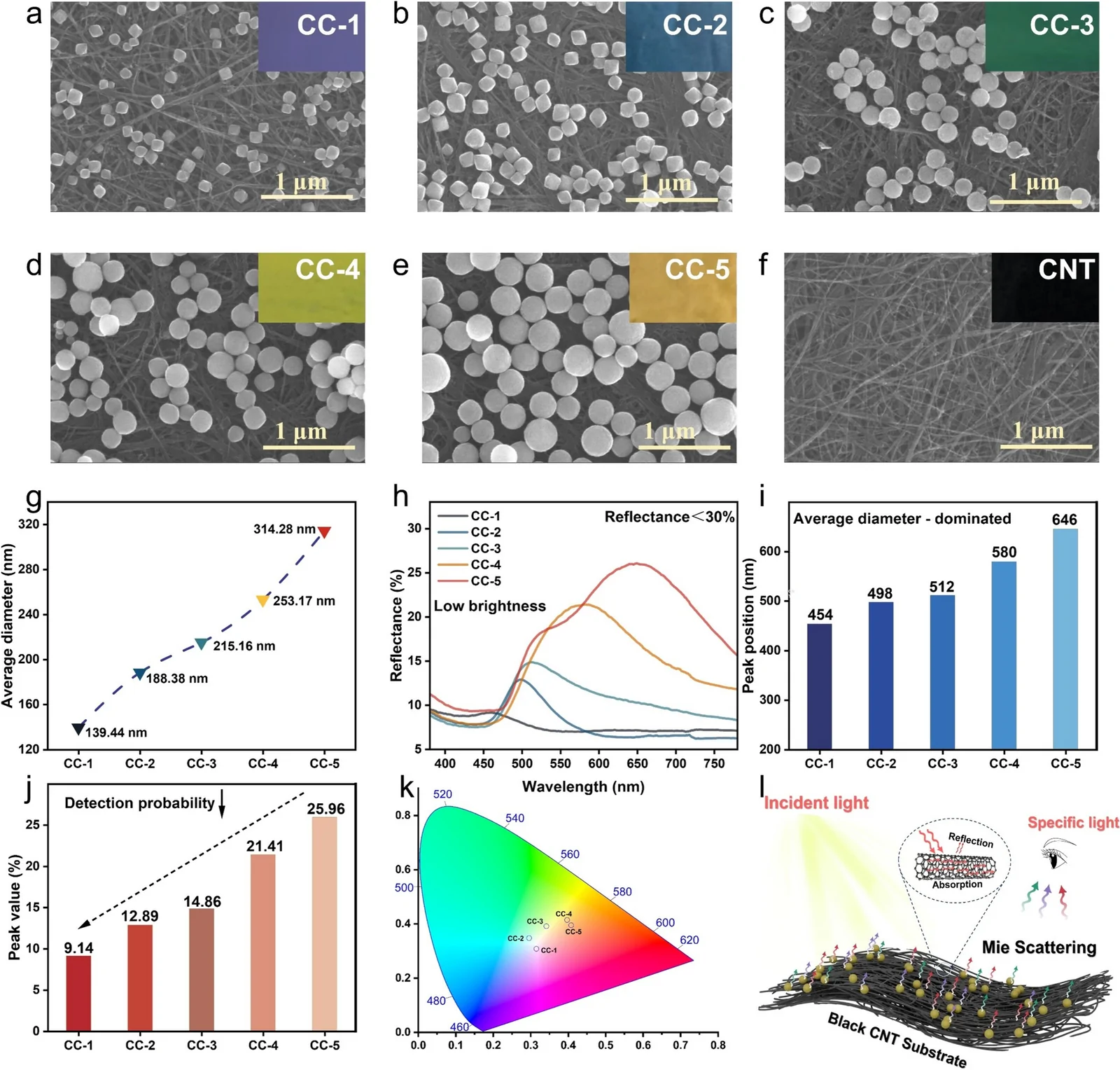
Morphological characterization, spectral curve, corresponding quantized data and coloration mechanism. SEM images of **a** CC-1, **b** CC-2, **c** CC-3, **d** CC-4, **e** CC-5 composite films, and **f** CNT film (illustration is the corresponding optical photograph.). **g** The average diameter of nanoparticles on the surface of the composite films. **h** Visible reflectance spectrum of the composite films. **i** Peak positions. **j** Peak value. **k** CIE 1931 chromaticity diagram. **l** The coloration mechanism of Cu_2_O nanoparticles on CNT film

According to the procedure detailed in Sects.  2.3 and 2.4, we successfully construct green CC-3 composite film with added Cu^2+^/citrate molar ratios of 1:1. Systematic modulation of molar ratios to 1:1.1 and 1:1.25 yields distinct yellow (CC-4) and orange (CC-5) films, respectively. SEM images confirm non-close-packed Cu_2_O nanospheres preserving the underlying bundled CNTs network architecture (Fig. 2c–e). Comparative pure CNT film morphology is presented in Fig. 2f. Energy-dispersive X-ray spectroscopy (EDS) elemental mapping demonstrates homogeneous distribution of C (96.29 at%), O (3.08 at%), and Cu (0.63 at%) in CC-3, with Cu species exhibiting disordered spatial arrangement complementary to carbon-rich domains (Fig. S4). To extend the color gamut toward blue/purple hues, citrate addition is reduced while maintaining PVP molecular weight (Mw = 58,000) and L-ascorbic acid dropping rate (3 mL min^−1^). Regrettably, merely controlling Cu^2+^/citrate ratios to 1:0.9, 1:0.8, and 1:0.65 uniformly yields composite films retaining the intrinsic black appearance of the CNTs substrate (Fig. S5d). SEM images reveal densely distributed Cu_2_O nanospheres with small diameter across these films (Fig. S5a–c). This morphological shift originates from reduced citrate additions elevating free Cu^2+^ concentration, thereby accelerating nucleation kinetics while curtailing nanoparticles growth duration. Increasing spray deposition frequency (> 40 passes) generates weak unsaturated yellow-green hues (Fig. S5d, e), attributable to intrinsic light absorption of close-packed Cu_2_O nanoparticles rather than structure color effects [38].

Based on above investigation, achieving broad spectral coverage necessitates precise co-regulation of nanoparticles dimensions and areal density. Thus, control experiments are conducted to explore related influencing factors. Maintaining Cu^2+^/citrate molar ratios of 1:0.8, switching to PVP with molecular weight of 55,000 produces green film. SEM image confirms nanosphere-dominated surfaces (about 200 nm, Fig. S6a), resulting from diminished steric hindrance [39] of shorter polymer chains facilitating particles growth (Fig. S6b). To further explore the influence of L-ascorbic acid dropping rate, we decrease dropping rate to 0.8 mL s^−1^, yielding yellow film (Fig. S6c), where slowed reduction kinetics initial nuclei populations. This promotes extensive particles growth via Ostwald ripening [40] while simultaneously inducing anisotropic crystal development through regulation of certain crystal plane, yielding nanoscale polyhedral morphologies (Fig. S6d). Both variables reduce packing density and increase particles diameter, enabling pronounced structure color emergence. Therefore, based on the research results of above control experiments, the process parameters are optimized. Further reducing the amount of citrate addition to initially control the particle diameter, and taking into account the spatial stereoscopic effect of PVP as well as the influence of the reduction rate, purple (CC-1) and blue (CC-2) composite films are successfully fabricated. SEM images confirm polyhedral-structured nanoparticles (Fig. 2a, b). The nanoparticles present disordered and non-closely dispersed state, analogous to that of CC-3/4/5 composite films. The key process parameters of the colorized composite films are shown in Table S1.

The diameter distributions of CC-1 to CC-5 surfaces are quantified in Fig. 2g with corresponding histograms (Fig. S7). Average diameters increase systematically from 139.44 nm (CC-1) to 314.28 nm (CC-5), establishing a nanoscale-mediated optical response where increasing dimensions induces progressive chromatic shifts. UV–Vis-NIR reflectance spectroscopy (380–780 nm, Fig. 2h) reveals characteristic peaks with intensities quantified in Fig. 2i, j. Systematic redshift of reflection peak position correlates with average diameter escalation, corresponding directly to visual transitions from violet to orange. Consistently low reflectance pattern confirms matte surface characteristics meeting military camouflage requirements. CIE 1931 chromaticity coordinates (Fig. 2k) and CIELAB parameters (Table S2) demonstrate comprehensive spectral control. Luminance (L*) increases from 1049.1155 (CC-1) to 1478.8144 (CC-5), and chromaticity parameters a* (−178.5935 to + 177.1464) and b* (−71.4013 to + 639.1973) also exhibit marked variations. This diameter-dependent optical response fundamentally differs from pigment-based coloration mechanisms, conclusively verifying successful implementation of structure color.

To assess the effectiveness of the composite films for visible camouflage, simulation camouflage experiments are implemented using CC-3 and CC-5 composite films. Uncoated pristine CNT films exhibit high visual detectability against grassland and soil backgrounds due to its intrinsic broadband absorption characteristics. In contrast, the green CC-3 composite film demonstrates effective spectral matching with vegetated environment, enabling visual blending in grassland scenarios. Similarly, orange CC-5 composite film achieves superior chromatic conformity with desertification-like soil background (Fig. S8). And the structure color presentation has excellent durability and environmental tolerance (Fig. S9).

Furthermore, a detailed investigation of the coloration mechanism of Cu_2_O is conducted. Assuming the color presentation is caused by the common Bragg diffraction (Eqs. 1 ~ 3) [41], it will conform to the model express as follows:

1 $$n_{{\mathrm{s}}}^{2} f_{{\mathrm{s}}} + n_{{{\mathrm{air}}}}^{2} (1 - f_{{\mathrm{s}}} ) = n_{{{\mathrm{eff}}}}^{2}$$

2 $$m\lambda_{max} = 2d_{hkl} \sqrt {\left( {n_{eff}^{2} - \sin \theta^{2} } \right)}$$

3 $$d_{hkl} = \frac{\sqrt 2 D}{{\sqrt {h^{2} + k^{2} + l^{2} } }}$$

Using established parameters [42, 43] *n*_s_ = 2.7 (refractive index), *f*_s_ = 0.74 (theoretical FCC packing fraction), *n*_air_ = 1.0, m = 1 (first-order diffraction), and *θ* = 0° (normal incidence), the theoretical diameter range is calculated as 116 ~ 239 nm (corresponding to 380 ~ 780 nm band). Even based on the SEM images, with f_s_ = 0.34, the theoretical particle size of Cu_2_O is 83 ~ 169 nm. Both theoretical results exhibit significant deviation from experimental measurement (100 ~ 400 nm). Furthermore, SEM images reveal disordered nanoparticles distributions lacking photonic crystal signatures (even short-range ordering of amorphous photonic crystal), this discrepancy definitively precludes Bragg diffraction as the primary coloration mechanism. Herein, Mie scattering theory (Eqs. 4 ~ 5) provides the reasonable framework [44]:

4 $$Q_{sca} = \frac{2}{{x^{2} }}\mathop \sum \limits_{n = 1}^{\infty } \left( {2n + 1} \right)\left( {\left| {a_{n} } \right|^{2} + \left| {b_{n} } \right|^{2} } \right)$$

5 $$x = \frac{2\pi r}{\lambda }$$

In this framework, the average diameter of Cu_2_O nanoparticles corresponds to a size parameter *x*. Its value falls within the range of 1 to 10 (380 ~ 780 nm), resulting in diminished dominance of forward scattering coupled with enhanced omnidirectional scattering. Thus, the angular distribution uniformity of scattered light intensity improves. This behavior aligns with experimentally observed low angular dependence in coloration phenomena, further confirming Mie scattering as the predominant coloration mechanism.

However, when Cu_2_O/ethanol dispersion (corresponding to the CC-3 composite film) is sprayed onto an A4 sheet of paper, no green structure color can be observed. Instead, the intrinsic color of Cu_2_O is displayed (Fig. S10a). Therefore, CNTs undoubtedly play a crucial role in the coloration process. Considering that CNTs have excellent electrical conductivity, other conductive substrates are used to investigate the influence of plasma resonance [45]. Alternative high-conductivity substrates (Al foil, Cu foil, Al sheet) exclusively exhibit the intrinsic orange-brown color of Cu_2_O (Fig. S10b-d). This observation eliminates the possibility of plasmonic coupling as an auxiliary mechanism for modulating light absorption. In contrast, black insulative substrates (black pasteboard, black cardboard) induce pronounced green color, corresponding to a distinct narrowband reflectance peak at 520 ± 10 nm (Fig. S10e, f). The Lab chromaticity coordinates of above-mentioned different colors are shown in Table S3. Above substrate experiments corroborate the essential role of optically black substrates in enabling structure coloration, arising from fundamental light-matter interactions. While Mie scattering generates wavelength-selective reflectance when nanoparticle dimensions approach visible light wavelengths, this effect becomes visually discernible only when transmitted light is quenched by an optically dense medium. The exceptional broadband absorbance of CNTs substrate effectively eliminates transmitted light and background reflections, thereby isolating the nanoparticle scattering signature. Conversely, on reflective or transparent substrates, broadband interference from transmitted/reflected light spectrally dilutes the scattered wavelengths, leaving only the inherent orange-brown hue from bulk bandgap absorption of Cu_2_O, logically consistent with the existing research to a high degree [46]. This substrate-dependent optical behavior underscores that structure coloration manifests not solely from nanoparticle properties, but from synergistic particle–substrate coupling where the absorber suppresses parasitic optical pathways to amplify resonant scattering (Fig. 2l).

### Macroscopic Adaptive IR Camouflage Performance and Revelation of Voltage-Response Mechanism

**Fig. 3:**
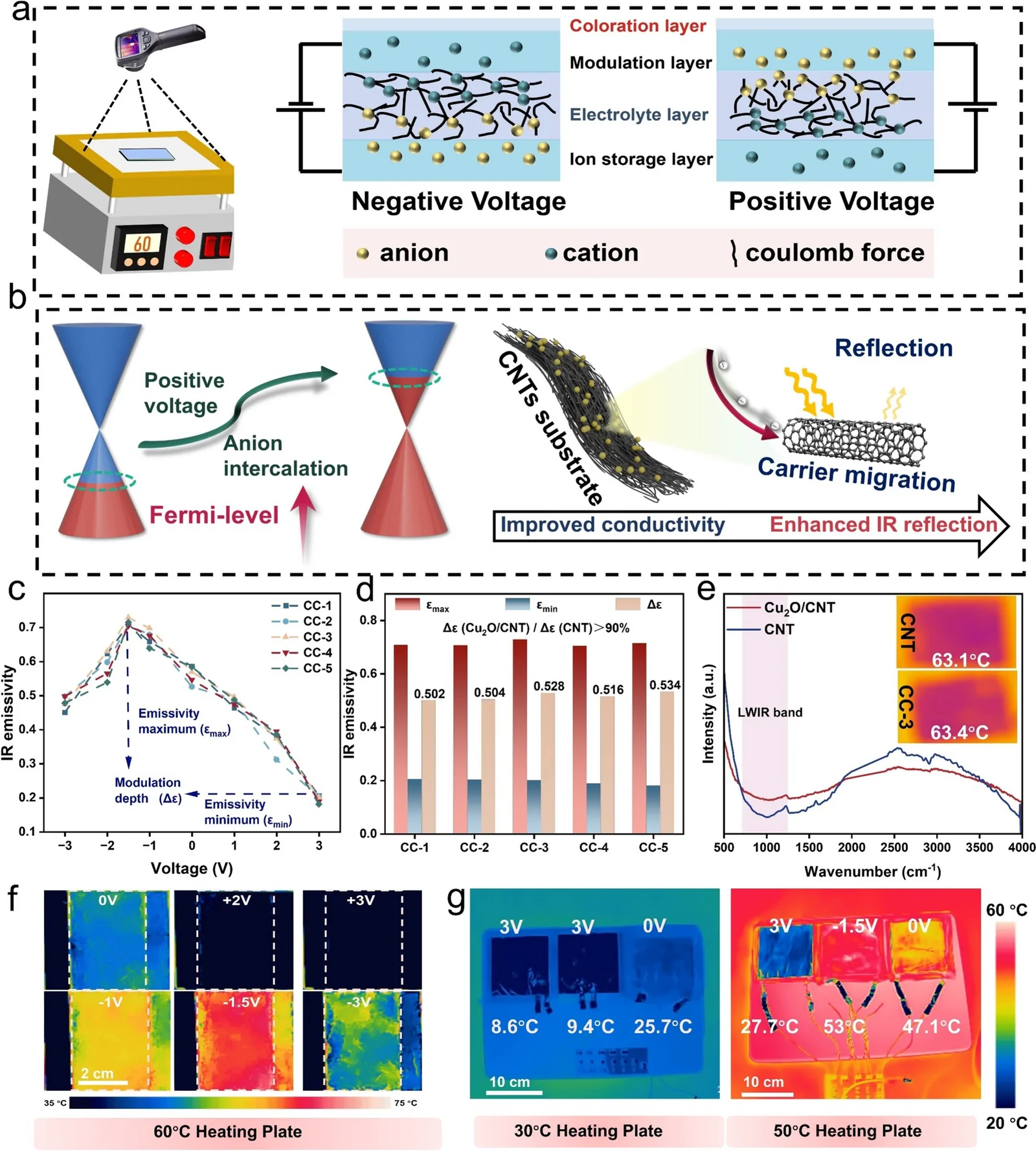
The infrared stealth performance and mechanism of composite films. **a** Schematic diagram of IR emissivity testing device and IR electrochromic device structure. **b** Voltage-response mechanism of IR emissivity. **c** The variation of the IR emissivity of composite films with voltage. **d** The maximum and minimum IR emissivity values and modulation depth of composite films. **e** FTIR transmittance spectra comparison and IR thermal image comparison between CNT film and CC-3 composite film. **f** IR thermal images of a single device under different voltages. **g** IR thermal images of 3 × 1 array under different voltages

Section 3.2 has confirmed the wide color gamut realization and the potential for visible camouflage of the Cu_2_O/CNT composite films. To further evaluate the adaptive IR camouflage performance, light sandwich-structured IR electrochromic devices are assembled with an areal density of 22.4 ± 0.4 mg cm^−2^ implemented on a 60 °C thermostatic platform (Fig. 3a). Cross-sectional SEM image clearly visualizes this structure (Fig. S11). When subjected to a bias voltage sweep from −3 to + 3 V, the ions in the electrolyte achieve reversible intercalation/deintercalation behavior. Crucially, IR emissivity reduction under positive bias voltage originates from Fermi-level elevation in CNTs induced by anion intercalation (Fig. 3b). This electrochemical process facilitates interfacial charge transfer, effectively increasing both carrier concentration and mobility within the CNTs. As shown in Fig. S12, the conductivity is tested for the CC-3 composite film system as a representative example. The consequent enhancement in electrical conductivity promotes IR reflectance while suppressing IR thermal emissivity, consistent with predictions from the Drude model (Eq. 6) [47]:

6 $$\varepsilon_{\left( \omega \right)} = 1 - \frac{{\omega_{p}^{2} }}{{\omega^{2} + {\raise0.7ex\hbox{${i\omega }$} \!\mathord{\left/ {\vphantom {{i\omega } \tau }}\right.\kern-0pt} \!\lower0.7ex\hbox{$\tau $}}}}$$

where ω_p_ is the plasma frequency and τ scattering time.

IR thermal images analysis reveals that the Cu_2_O/CNT composite films demonstrate remarkable response characteristics in the LWIR band, achieving IR modulation depth (Δε) exceeding 0.5 with overall emissivity consistently below 0.75. Specifically, Δε for CC-1/2/3/4/5 devices are measured as 0.502 (0.708 ~ 0.206), 0.504 (0.707 ~ 0.203), 0.528 (0.729 ~ 0.201), 0.516 (0.705 ~ 0.189), and 0.534 (0.715 ~ 0.181), respectively (Fig. 3c, d). To evaluate the stability of the devices, an environmental tolerance test (85 °C temperature/85% relative humidity) and a 600-cycle charge–discharge test (CC-3 as the representative) are conducted, and the modulation depth remains high, demonstrating practical potential for long-term application (Fig. S13).

The modulation depths of Cu_2_O/CNT composite films consistently reach over 90% of that of the original CNT film, demonstrating IR transparency of Cu_2_O nanoparticles. Besides aforementioned intrinsic transmittance across LWIR band (FTIR spectral congruence and IR images similarity between CNT film and CC-3 composite film further confirms negligible IR absorption, Fig. 3e), the overall submicron-scale thickness (< 1 μm) and non-close-packed Cu_2_O architectures also permit IR optical transmission. The influence of thickness can be described through Lambert-Bouguer Law (Eq. 7) [48]:

7 $$\tau_{i} \left( \lambda \right) = \tau_{{i_{0} }} \left( \lambda \right){l \mathord{\left/ {\vphantom {l {l_{0} }}} \right. \kern-0pt} {l_{0} }}$$

where ${\tau }_{{\mathrm{i}}_{0}}\left(\lambda \right)$ denotes the spectral transmittance at a certain wavelength, ${l}_{0}$ represents reference thickness, l is the thickness of the selected material. Therefore, the transmittance within IR spectrum increases as the thickness of the material through which the radiation passes decreases.

Figure 3f provides an intuitive illustration of the IR thermal images of the CC-3 composite film with respect to the bias voltage. At −1.5 V, the IR thermal image appears as red, corresponding to a high IR emissivity state. At + 3 V, the IR thermal image shows as dark blue, corresponding to a low IR emissivity state. The IR thermal image can undergo significant changes with a response time of less than 2 s. Figure 3g demonstrates a 3 × 1 array (10 × 10 cm individual elements) subjected to differential bias voltages. Under isothermal conditions at 30 °C, application of + 3 V to the first two elements reduces their IR temperatures to 9 ± 0.4 °C, while the unbiased third element maintains 25.7 °C. When transferred to a 50 °C thermal platform with sequential biasing (+ 3, −1.5, and 0 V), the devices exhibit corresponding IR temperatures of 27.7, 53, and 47.1 °C, sustaining a maximum thermal contrast of 25.3 °C. Through modular control, the “NUAA” characters (abbreviation of Nanjing University of Aeronautics and Astronautics) are "written" in the LWIR band (Fig. S14). Figure S15 further demonstrates the excellent mechanical properties.

### Electrothermal Energy Conversion Dynamics and Application Efficacy Presentation

Cu_2_O/CNT composite films have been confirmed the potential to achieve wide color gamut coverage and dynamic IR emissivity. According to the Boltzmann Stefan-Boltzmann law (*P* = εσT^4^) [49, 50], the IR radiation characteristic also depends on the regulation of temperature to meet more military requirements and backgrounds. Since Cu_2_O nanoparticles and CNT film are only physically coupled at the interface, it is encouraged to further explore the potential electrothermal energy conversion performance of Cu_2_O/CNT composite films.

**Fig. 4:**
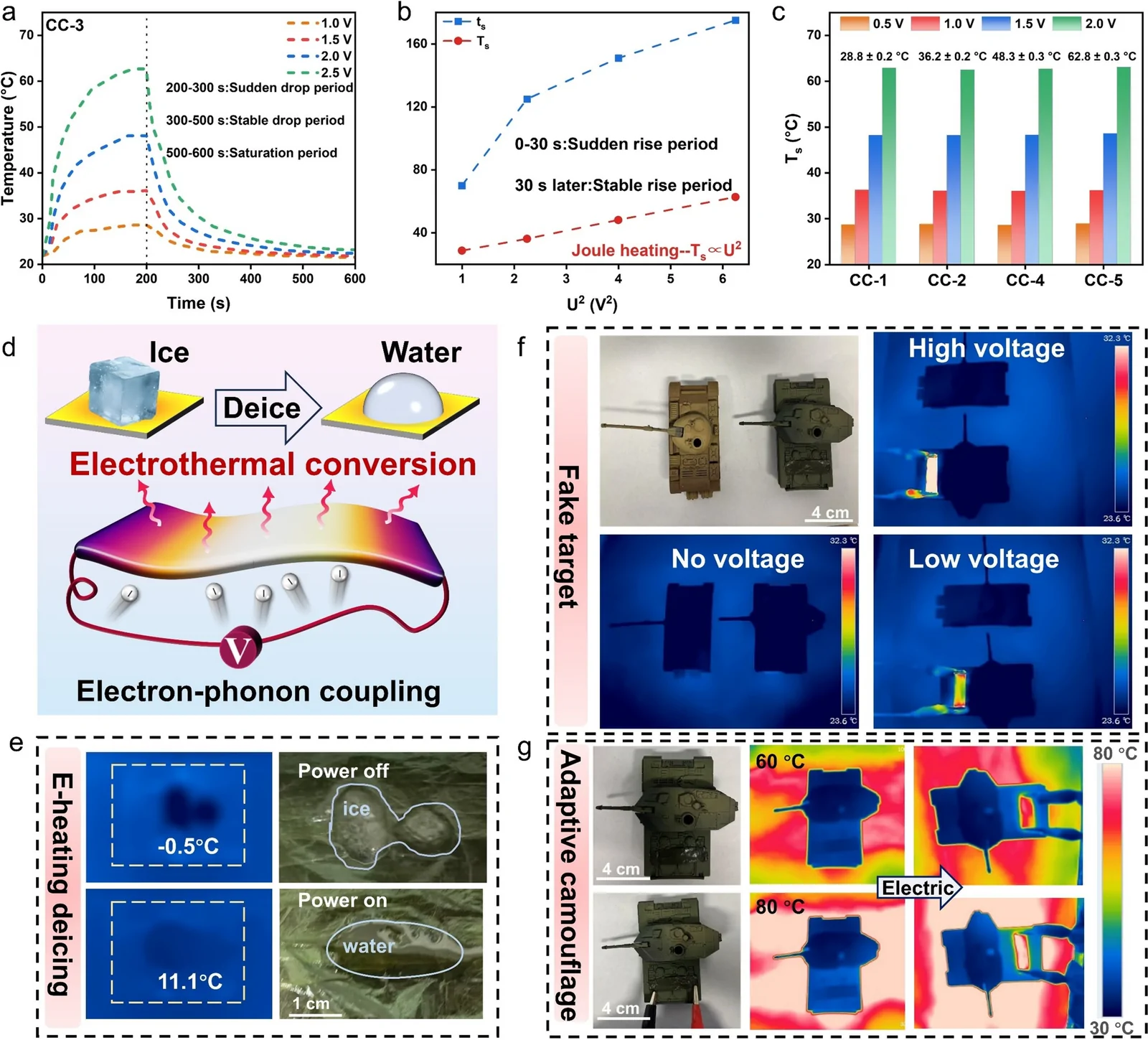
Electrothermal energy conversion characteristic and application scenarios of composite films. **a** Temperature changes corresponding to the voltage of CC-3 composite film. **b** Saturation temperature (T_S_), temperature saturation time (t_s_) of CC-3 composite film under different voltages. **c** Saturation temperatures of other composite films (CC-1/2/4/5) under different voltages. **d** Schematic diagram of the electrothermal conversion mechanism. **e** Digital photograph and IR thermal images of deicing. **f** IR disguise of the fake target. **g** Adaptive equipment photographs and IR camouflage of a low-temperature target in simulating different high-temperature backgrounds

Comprehensive energy conversion under applied DC voltages (1.0 ~ 2.5 V) demonstrates rapid heating kinetics in CC-3 composite film, where saturation temperature (T_S_) follows distinct voltage-dependent trend. As the voltage increases, the time (t_S_) needed to reach T_S_ will also prolong. This trend can be understood by heat transfer dynamics and material responses (Figs. 4a, b and S16a, b). These systematic variations during the 200-s activation phase reflect fundamental charge transport phenomena within the CNT film. This hierarchical thermal response originates from efficient electron–phonon coupling [51] within the percolating CNTs network, where accelerated charge carriers transfer kinetic energy to the lattice through inelastic scattering, transforming into heat energy (Fig. 4d). Upon voltage cessation, thermal relaxation exhibits triphasic decay kinetics—an initial rapid cooling phase (200 ~ 300 s) governed by convective heat dissipation to ambient air, followed by gradual temperature reduction (300 ~ 500 s) dominated by conductive loss through substrate interfaces, ultimately reaching thermal equilibration (500 ~ 600 s) governed by steady-state radiative exchange. Importantly, the observed characteristic linear relationship between saturation IR temperature and applied voltage squared (T_S_ is approximately proportional to U^2^) fundamentally validates Joule heating dominance according to classical electrothermal principles [52]. Consistent electrothermal behavior across all composite films evidences CNTs-dominated energy conversion mechanisms (Fig. 4c), with identical dynamics further confirming unimpeded electron transport through the underlying CNTs network. Furthermore, the cyclic stability of the composite film in a high humidity environment (85% RH) demonstrates the application potential (Fig. S16c).

Due to the ideal electrothermal conversion performance, when an external voltage of 2.5 V is applied, the frozen ice on CC-4 composite film can completely melt within 30 s. The corresponding IR images confirm the ice melting process from −0.5 to 11.1 °C on the surface, which demonstrates the potential application of Cu_2_O/CNT composite films for deicing (Fig. 4e). Besides, by applying an external electric field, the surface IR radiation temperature of composite films can be rapidly adjusted to match the IR radiation characteristics of non-target objects or fake radiation signals, thereby achieving IR camouflage for more scenarios. As shown in Fig. 4f, the CC-4 composite film can be designed as distinguishable fake targets through instantaneous IR thermal characteristics replication to achieve the effect of intentional deception. It is shown in the IR image as yellow-green and even pink-white fake target standing out against the dark blue background due to such nearly instantaneous response, causing a credible deception to the IR surveillance system. Construction of fake targets is crucial for consuming the combat power of enemies and helping the real targets avoid the risk of being attacked. More practically, military equipment inevitably encounters rapidly changing combat environments [53]. When transitioning from a cold environment to a hot environment, simple control of IR emissivity alone has limitations [54]. As shown in Fig. 4g, certain part of the tank covered by CC-4 composite film can achieve adaptive IR camouflage proved by excellent integration at both 60 and 80 °C backgrounds (simulation of the desertification environment in certain areas).

The composite films additionally demonstrate robust photothermal conversion capabilities under simulated solar irradiation (100 mW cm^−2^ xenon lamp). The CC-3 composite film achieves thermal saturation at 88.4 °C within 52 s, whereas the pristine CNT film attains 107.7 °C in 45 s (Fig. S17a, b). This moderated photothermal efficiency arises from the Cu_2_O-induced enhancement in spectral reflectance, which increases from 8% to 16% across the 780 ~ 2500 nm band (Fig. S17c). The nanoparticles integration introduces supplementary photon reflection pathways while moderately reducing broadband absorption, thereby diminishing IR radiation generation. Despite such tiny attenuation, the retained high photothermal conversion capability still satisfies the requirement of most operations in high-temperature environments [55], establishing a complementary control dimension to the electrothermal functionality.

### Enhanced EMI Shielding Performance and Mechanism Revelation

The EMI shielding performance is of great significance for preventing the leakage of military information and protecting the integrity of electronic components in equipment [56–58]. Thus, this work uses a vector network analyzer to investigate the interaction mechanism between the incident wave and sandwich-structured Cu_2_O/CNT/IL/CNT device (denoted CC-n device), and further optimize the EMI shielding performance via densifying the conductive network of CNT film and increasing defect sites induced by functional groups while ensuring that the IR modulation width is basically unaffected. According to electromagnetic theory (Eqs. S1-S7), the EMI SE of materials is mainly related to real thickness and electrical conductivity. The total EMI SE (SE_T_) is the sum of the absorption (SE_A_) within the material, multiple reflections at different interfaces (SE_M_), and reflections at the surface (SE_R_) [59]. Since SE_T_ is greater than 10 dB, the influence of multiple reflections can be excluded [60].

**Fig. 5:**
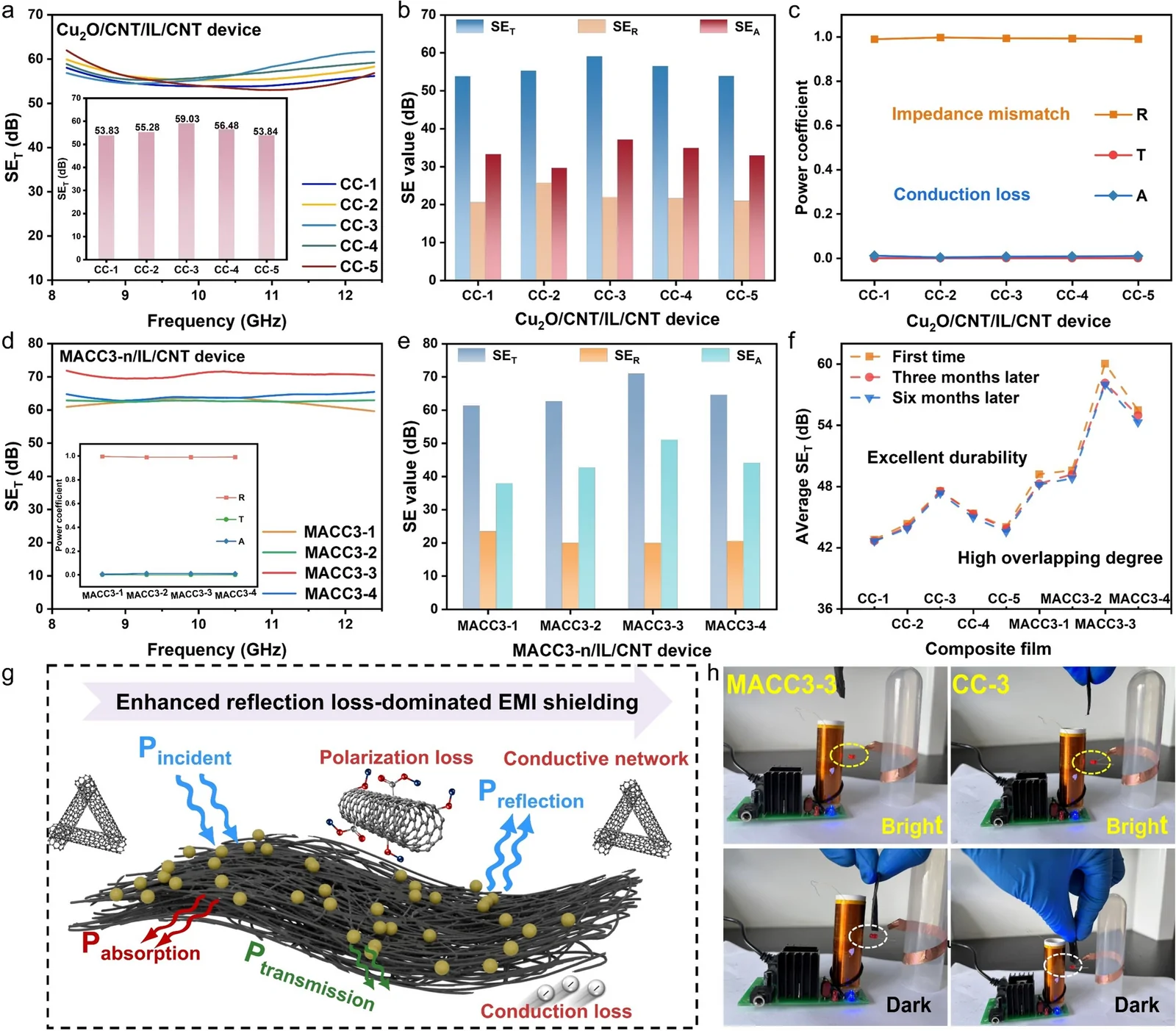
Electromagnetic interference (EMI) shielding effectiveness and mechanism. CC-1/2/3/4/5 devices **a** Variation of the SE_T_ values with frequency. **b** Average SE_T,_ SE_R,_ SE_A_ values. **c** Power coefficients A, R, and T. MACC3-1/2/3/4 devices **d** Variation of the SE_T_ values with frequency (inset is illustration of power coefficients A, R, and T). **e** Average SE_T,_ SE_R,_ SE_A_ values. **f** Long-term stability test of the shielding effectiveness value. **g** Schematic diagram of the EMI shielding mechanism of the MACC3 composite films. **h** Bulb conditions variation before and after inserted into MACC3-3 and CC-3 composite films

As shown in Fig. 5a, the CC-n devices demonstrate exceptional EMI shielding effectiveness (53 ~ 60 dB) across the X-band (8.2 ~ 12.4 GHz). Figure 5b reveals the synergistic contribution of reflection and absorption losses. Power coefficient analysis (reflectivity R, absorptivity A, transmissivity T) in Fig. 5c indicates R≈1, confirming reflection loss as the dominant mechanism [61]. This originates from impedance mismatch induced by the high conductivity of CNT film, causing significant wave reflection. Compared to the device composed of pure CNT film (denoted CNT device), CC-n devices exhibit marginally reduced SE_T_ value, primarily due to decreased absorption loss, while reflection loss remains comparable. This trend persists in direct comparisons between CNT film and Cu_2_O/CNT composite film (Fig. S18). The minimal thickness of Cu_2_O nanoparticles likely preserves interface impedance, yet their partial coverage on CNTs disrupts conductive network continuity, reducing conduction loss. Even placed at a relatively extreme condition of 85 °C/85%RH for one hour, the CC-n devices still maintain excellent EMI shielding performance, showing excellent tolerance (Fig. S19).

To enhance shielding effectiveness, CNT films are functionalized via mixed-acid surface grafting (denoted MACNT-1/2/3/4 films). Subsequent deposition of Cu_2_O nanoparticles (CC-3 formulation) yields green functionalized film (denoted MACC3-1/2/3/4 composite films). Devices composed of MACC3-n composite films (denoted MACC3-n device) exhibit superior EMI shielding efficacy, with MACC3-3 device achieving SE_T_ value of 70.97 dB, a 20% improvement over CC-3 device. Crucially, these devices retain effective color presentation, outstanding IR modulation depth (e.g., MACC3-3: Δε = 0.510) and even better electrothermal conversion ability, confirming unimpaired multifunctionality (Fig. S20a-e). Shielding mechanisms of MACC3-n devices are probed by analyzing SE_T_, SE_A_, and SE_R_ contributions alongside power coefficients (Fig. 5d, e). Performance remains governed by wave absorption and reflection, with high R values indicating persistent reflection dominance. Shielding effectiveness correlates positively with conductivity of functionalized composite films (Fig. S20f). Upon wave incidence, the percolative conductive network reflects most wave. Notably, SE_A_ values (37.89 to 51.07 dB) for MACC3-1/2/3 devices substantially exceed SE_R_ values (23.42 to 19.9 dB). This SE_A_ enhancement stems from reduced contact resistance improving conduction loss and dipole polarization loss at functional group-induced defect sites [62] (Fig. 5g). The inverse trend of MACC3-4 composite films arises from partial sp^2^-conjugation disruption.

Component-specific contributions are evaluated by comparing individual monolayer films with their sandwich-structured counterparts. Layered devices exhibit about 10 dB higher SE_T_ than corresponding composite films (Figs. S21 and S22), attributable to extended electromagnetic wave propagation paths enhancing absorption. Besides, unreflected waves undergo secondary reflection at interfaces. It is also found that the IL-immersed PE separator exhibits negligible shielding (SE_T_ = 0.0661 dB, T = 0.9849), confirming electromagnetic transparency (Fig. S23a). Similarly, Cu_2_O nanoparticles sprayed onto PE separator remain electromagnetic-transparent (SE_T_ = 0.1507 dB, T = 0.9665) (Fig. S23b). Furthermore, we conduct a quantitative study on the role of Cu_2_O in this system in terms of electromagnetic shielding (Figs. S24 and S25a-c). Finally, it is believed that the shielding performance of Cu_2_O/CNT/IL/CNT device primarily arises from reflection at top/bottom CNT layers (Fig. S25d). To verify their long-term stability, the above-mentioned nine composite films (CC-1 ~ 5, MACC3-1 ~ 4 composite films) are placed for three and six months. It can be observed that they greatly maintain the integrity of EMI shielding (Fig. 5f). Although their thin structure (Fig. S26a, b) poses a challenge, this remarkable stability still exists. The actual EMI shielding efficacy is clearly demonstrated through an induction experiment using a Tesla coil (Fig. 5h). Its working principle is shown in Fig. S26c [42]. The unshielded circuit keeps the bulb illuminated through electromagnetic induction, while inserting the CC-3, MACC3-3 films instantly extinguishes the bulb. The other composite films also exhibit the same performance. In contrast, A4 paper could not extinguish the bulb when inserted (Fig. S26d).

Table S4 presents the effective integration compared with other military defense systems. A quantitative comparison with other reported military defense systems is also made regarding rich coloration, IR modulation width, EMI interference shielding effectiveness (Fig. S27). The multi-scale optical engineering system demonstrates excellent performance and multifunctional integration characteristics (Fig. S28), providing an important paradigm for cross-band camouflage technologies.

## Conclusions

In summary, this work establishes a multi-spectral camouflage system through the design of Cu_2_O/CNT/IL/CNT-layered structure. Using Cu_2_O nanoparticles with Mie scattering to achieve wide color gamut coverage while maintaining IR transmittance, the dynamic IR modulation performance of the CNTs is well preserved. This structure overcomes the traditional trade-off between color richness and dynamic IR radiation control with fast response time as well as excellent cycle stability. The preserved CNTs network due to the interface physical coupling of Cu_2_O maintains excellent conductivity, achieving efficient electrothermal conversion. Crucially, through enhanced conduction loss and polarization loss induced by oxygen-containing functional group, exceptional EMI shielding is achieved. This work integrates multifunction into a lightweight composite material system, providing a design paradigm for military camouflage materials that necessitate simultaneous realization of visible-IR camouflage, EMI shielding protection, and thermal management in constantly changing battlefield environments.

## Supplementary Information

Below is the link to the electronic supplementary material.

Supplementary file1 (DOCX 5179 KB)
