# Supplementary material for: A Multi-Scale Cross-Band Defense System Integrating Decoupled Visible, Dynamic Infrared Camouflage and Electromagnetic Shielding
Source: Nanomicro Lett. 2026 Jan 13;18:115. doi: 10.1007/s40820-025-01961-4 (PMC12796066; doi:10.1007/s40820-025-01961-4)
Supplement: Supplementary file 1 — Supplementary file1 (DOCX 5179 KB) [file 40820_2025_1961_MOESM1_ESM.docx]

Supporting Information for

**A Multi-Scale Cross-Band Defense System Integrating Decoupled Visible, Dynamic Infrared Camouflage and Electromagnetic Shielding**

Junlin Liu^1^, Shujuan Tan^1^*, Xinrui Yang^1^, Jiajie Zhu^1^, Xin Yan^1^, Tianyu Chen^1^, Guangbin Ji^1^*

^1^ College of Material Science and Technology, Nanjing University of Aeronautics and Astronautics, Nanjing 210016, P. R. China

*Corresponding authors. E-mail: [tanshujuan@nuaa.edu.cn](mailto:tanshujuan@nuaa.edu.cn) (Shujuan Tan); [gbji@nuaa.edu.cn](mailto:gbji@nuaa.edu.cn) (Guangbin Ji)

**Note S1 Dynamic Infrared Response Measurement**

IR thermography records surface radiation temperatures (T_IR_) under applied voltages (Samples are placed on a constant temperature heating platform), while true temperatures (T_R_) are calibrated using 3M Scotch tape (ε = 0.95). Ambient temperature (T_0_) is monitored by thermocouples. Integrated IR emissivity (ε) is calculated by the following formula [S1-S3] :

$\varepsilon=\frac{T_{\mathrm{IR}}^{4}-T_{0}^{4}}{T_{R}^{4}-T_{0}^{4}}$ (S1)

The emissivity modulation depth (Δε) is defined as the difference between ε_max_ and ε_min_. The larger Δε is, the better the tunability of the IR radiation signal becomes. As voltage is applied, the color of the IR thermography will change. The time required for the color change area to stabilize is defined as response time.

**Note S2 Electromagnetic Interference Shielding Measurement**

The total electromagnetic interference shielding effectiveness (SE_T_) is the sum of absorption loss (SE_A_) and reflection loss (SE_R_), as shown in the following formula [S4] :

SE_T_ = SE_A_ + SE_R_  (S2)

First, the S parameters of the X-band are obtained through waveguide method using a vector network analyzer. Then, based on the obtained S parameters, the reflection coefficient (R) and transmission coefficient (T) can be calculated by the following formulas:

$R=\left| s_{11} \right|^{2}$ （S3）

$T=\left| s_{21} \right|^{2}$ （S4）

Since the sum of the absorption coefficient (A), reflection coefficient and transmission coefficient is 1, so A can be calculated by the following formula:

A=1-R-T （S5）

Further calculation of SE_R_ and SE_A_ is shown in the following formulas:

$\mathrm{SE}_{R}=10\log\left( \frac{1}{1-R} \right)$ （S6）

$\mathrm{SE}_{A}=10\log\left( \frac{1-R}{T} \right)$ （S7）

**Table S1** Synthesis parameters of colorized composite films

| Sample | Na_3_C_6_H_5_O_7_.2H_2_O (g) | PVP | Dropping rate (mL/min) |
| --- | --- | --- | --- |
| CC-1 | 0.60 | Mw=55000 | 0.8 |
| CC-2 | 0.65 | M_W_=55000 | 0.8 |
| CC-3 | 1.00 | Mw=58000 | 3 |
| CC-4 | 1.10 | Mw=58000 | 3 |
| CC-5 | 1.25 | Mw=58000 | 3 |

**Table S2** Lab chromaticity parameters of composite films

| Sample | L* | a* | b* |
| --- | --- | --- | --- |
| CC-1 | 1049.1155 | 111.4974 | -71.4013 |
| CC-2 | 1111.7886 | -178.5935 | 39.3901 |
| CC-3 | 1264.3027 | -155.9012 | 319.6779 |
| CC-4 | 1448.9854 | 13.6808 | 639.1973 |
| CC-5 | 1478.8144 | 177.1464 | 592.2988 |

**Table S3** Lab chromaticity parameters of different substrates coating with Cu_2_O nanoparticles (CC-3 formulation)

| Substrate | L* | a* | b* |
| --- | --- | --- | --- |
| A4 paper | 2284.6087 | 187.9981 | 492.8160 |
| Al foil | 2305.7064 | 247.8245 | 245.2513 |
| Cu foil | 2055.5920 | 608.5176 | 608.2674 |
| Al sheet | 1634.8857 | 290.3930 | 340.5754 |
| Pasteboard | 916.4862 | -156.3869 | 186.0822 |
| Cardboard | 1066.4871 | -28.8304 | 108.5950 |

**Table S4** The multidimensional comparison with other reported military defense systems

| Material systems | External stimulus | Infrared modulation | Chromaticity richness | Function expansion |
| --- | --- | --- | --- | --- |
| Metal/Al_2_O_3_/Al [S5] | No. | Not achieved. | Achieved. | Not achieved. |
| Micro-nano SiO_2_@Al [S6] | No. | Not achieved. | Not achieved. | Not achieved. |
| MXene/LiClO_4_/MXene [S7] | Electric | Achieved. | Not achieved. | Not achieved. |
| Pigment/CNT [S3] | Electric | Achieved. | Achieved. | Not achieved. |
| Cr_2_O_3_@ATO [S8] | No. | Achieved. | Not achieved. | Radar stealth. |
| VO_2_/GR/CNT [S9] | Thermal | Achieved. | Not achieved. | Not achieved. |
| Cu_2_O/CNT (This work) | Electric | Achieved. | Achieved. | EMI shielding;  Energy conversion. |

In the two-step reduction method for preparing Cu_2_O nanoparticles, each substance plays its own specific role. Copper (II) acetate monohydrate serves as the copper precursor. Trisodium citrate dihydrate is used as complexing agent to prevent the precipitation of Cu(OH)_2_. By controlling the concentration of free Cu^2+^, the nucleation rate can be slowed down. PVP can modulate crystal faceting through selective polymer-plane interactions. L-Ascorbic acid plays a key role in sequential reduction of Cu^2+^ to Cu^+^. This stepwise reductant addition promotes uniform nucleation and growth kinetics of Cu_2_O crystallites. Premature complete reduction would induce instantaneous nucleation, leading to heterogeneous particles growth and uneven diameter distribution.

During the reaction process, the liquid phase system undergoes a series of color changes. The initial precursor solution containing Cu(CH_3_COO)_2_·H_2_O and C_6_H_5_Na_3_O_7_·2H_2_O appears transparent pale blue (Fig. S1a). Upon NaOH addition, the system transforms into a deep blue transparent state under alkaline conditions **(**Fig. S1b). Gradual infusion of L-ascorbic acid induces sequential color transitions of dark green to dull yellow (Fig. S1c-f). Subsequent secondary L-ascorbic acid supplementation yields a bright yellow solution (Fig. S1g).

**
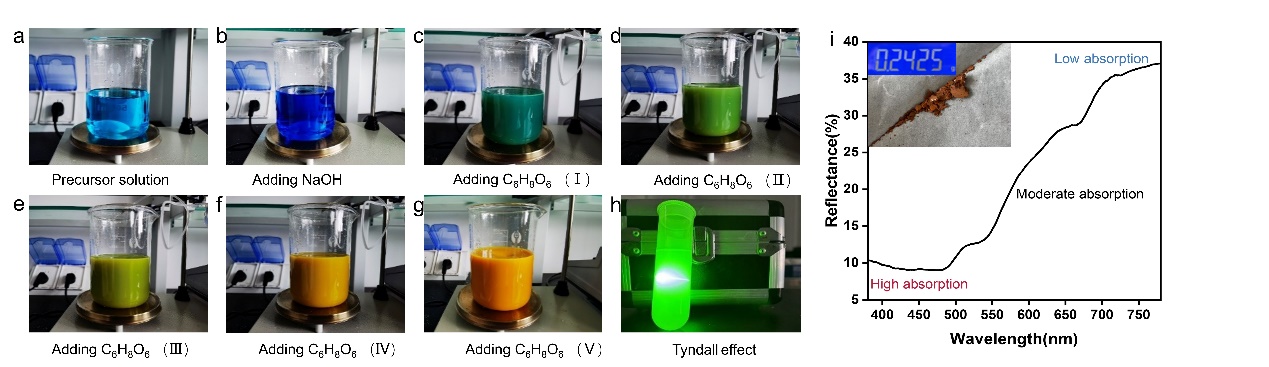
**

**Fig. S1 a-g** Color transitions of the solution during the synthesis process of Cu_2_O nanoparticles. **h** Tyndall effect of a low-concentration Cu_2_O/ethanol dispersion solution. **i** Spectral reflectance (380~780 nm) of Cu_2_O nanoparticles (Illustration is digital photograph and yield)

CNT films are synthesized via floating catalyst chemical vapor deposition (FFCVD) method (Fig. S2a), demonstrating intrinsic hydrophobicity (contact angle: 128 ± 2°, Fig. S2b). Reflectance spectroscopy (380–2500 nm, Fig. S2c) reveals low reflectivity (≈8%), consistent with macroscopic black appearance. The atomic force microscopy (AFM) images show that the CNT film prepared has a certain degree of roughness (Fig. S2d).

**
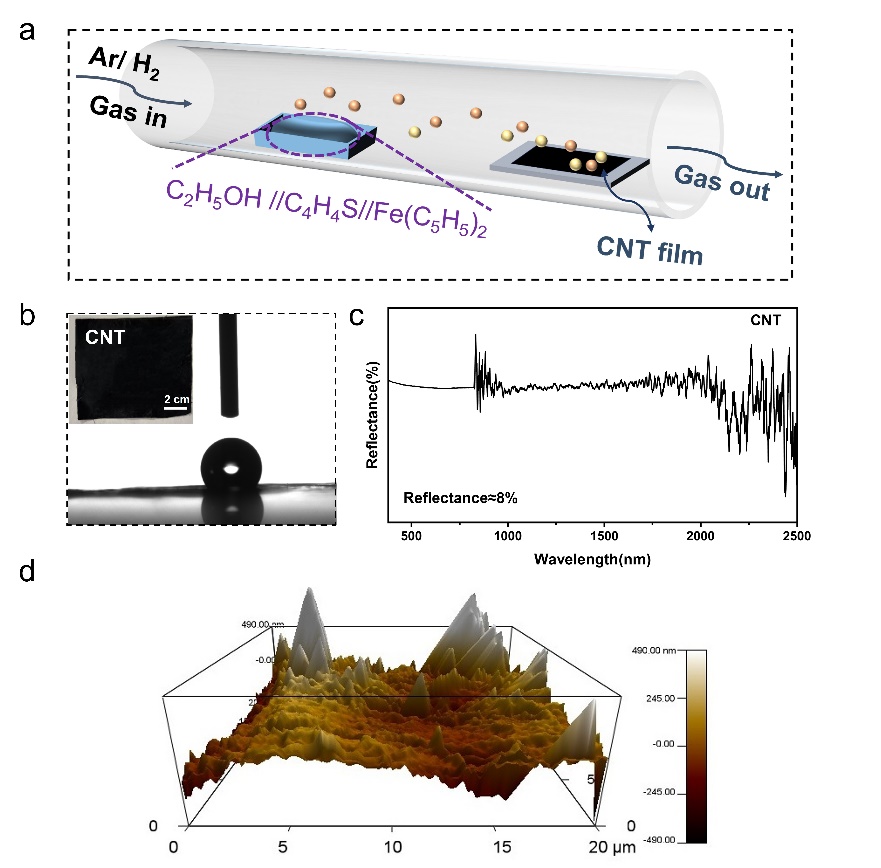
**

**Fig. S2 a** The process of preparing CNT films by FFCVD method. **b** Digital photograph of CNT film and illustration of hydrophobicity. **c** The VIS-NIR reflectance spectrum of CNT film. **d** AFM image of CNT film


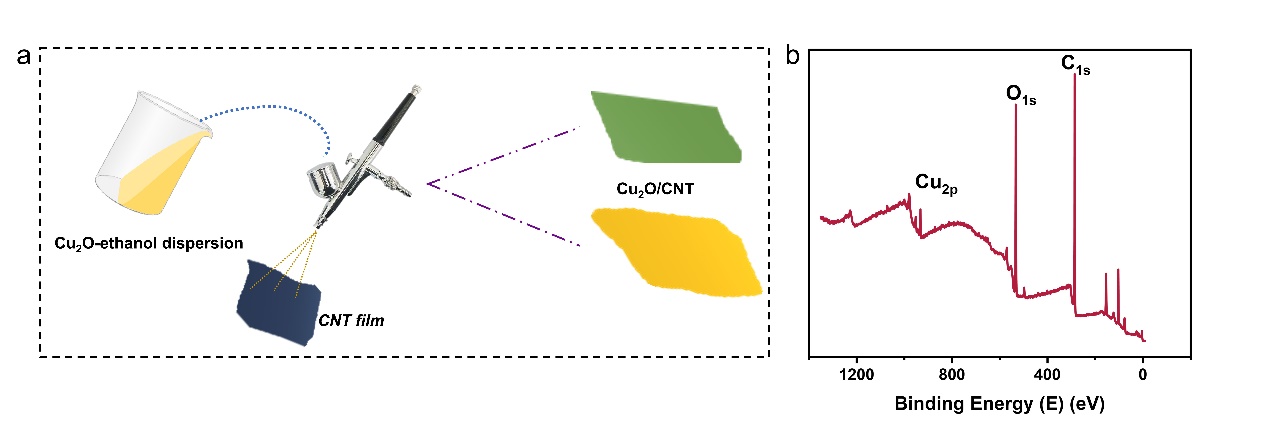


**Fig. S3** **a** Schematic diagram of preparing Cu_2_O/CNT composite films by air compression spraying method. **b** Total XPS spectrum of the CC-3 composite film


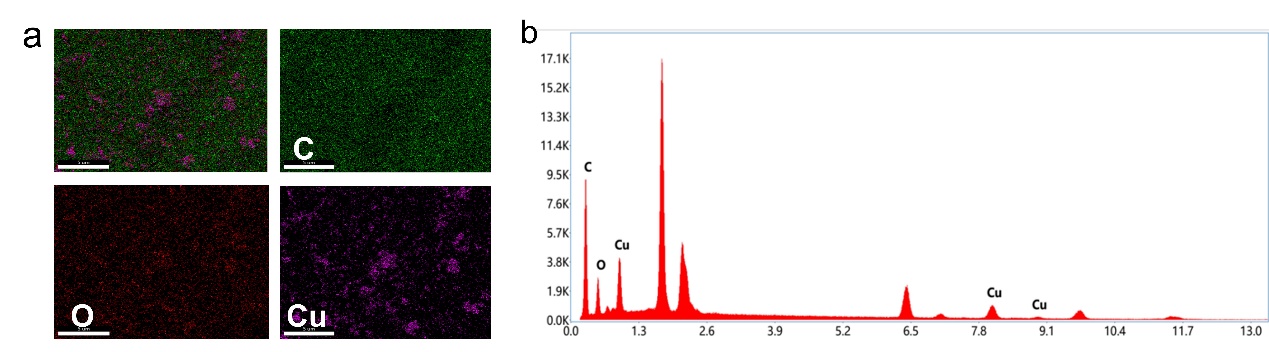


**Fig. S4** **a** EDS surface scan of CC-3 composite film. **b** Quantitative diagram of element distribution

**
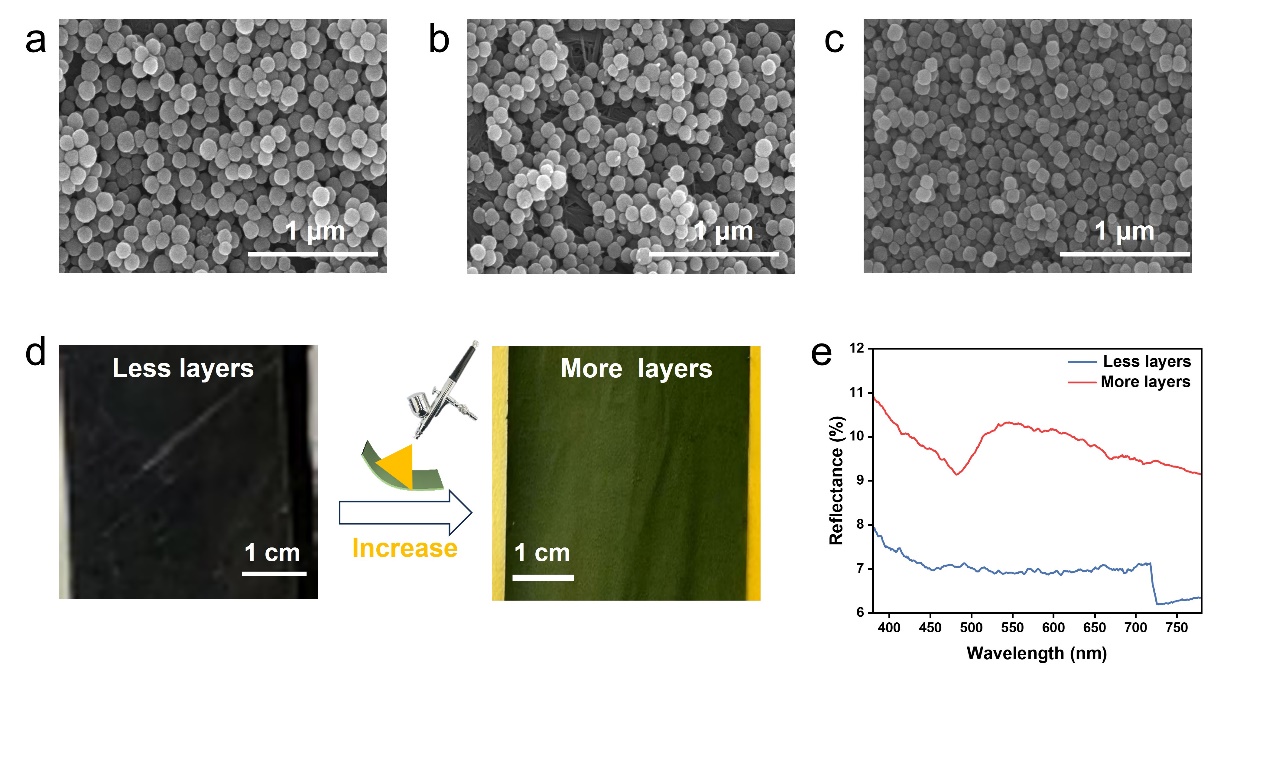
Fig. S5** **a-c** SEM images of the composite films obtained with Cu^2+^ to citrate molar ratios of 1:0.9, 1:0.8, and 1:0.65 (PVP of Mw=58000, ascorbic acid dropping rate of 3 mL/min). **d** Comparison of digital photographs of composite films obtained by different spraying layers. **e** Reflectance spectrum (380~780 nm) of the composite films in **d**.


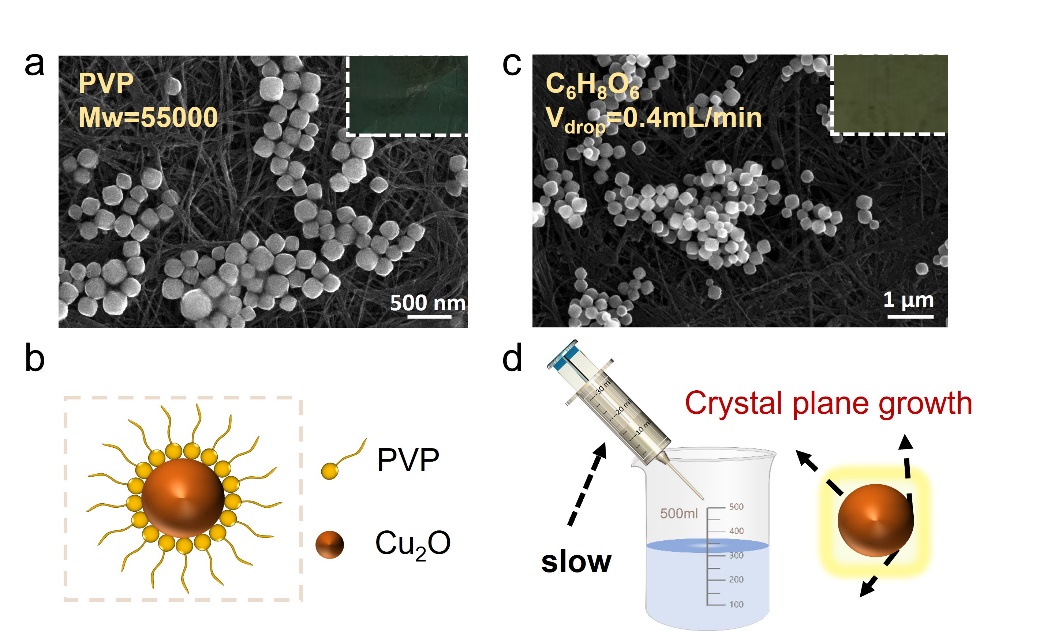


**Fig. S6** Molar ratio of Cu^2+^ to citrate is 1:0.8 **a** SEM image and digital photograph of the composite film (PVP with Mw=55000). **b** Schematic diagram of the PVP action mechanism. **c** SEM images and digital photograph of the composite film (the dropping rate of L-ascorbic acid is 0.4 mL/min). **d** Schematic diagram of the effect of L-ascorbic acid dropping rate


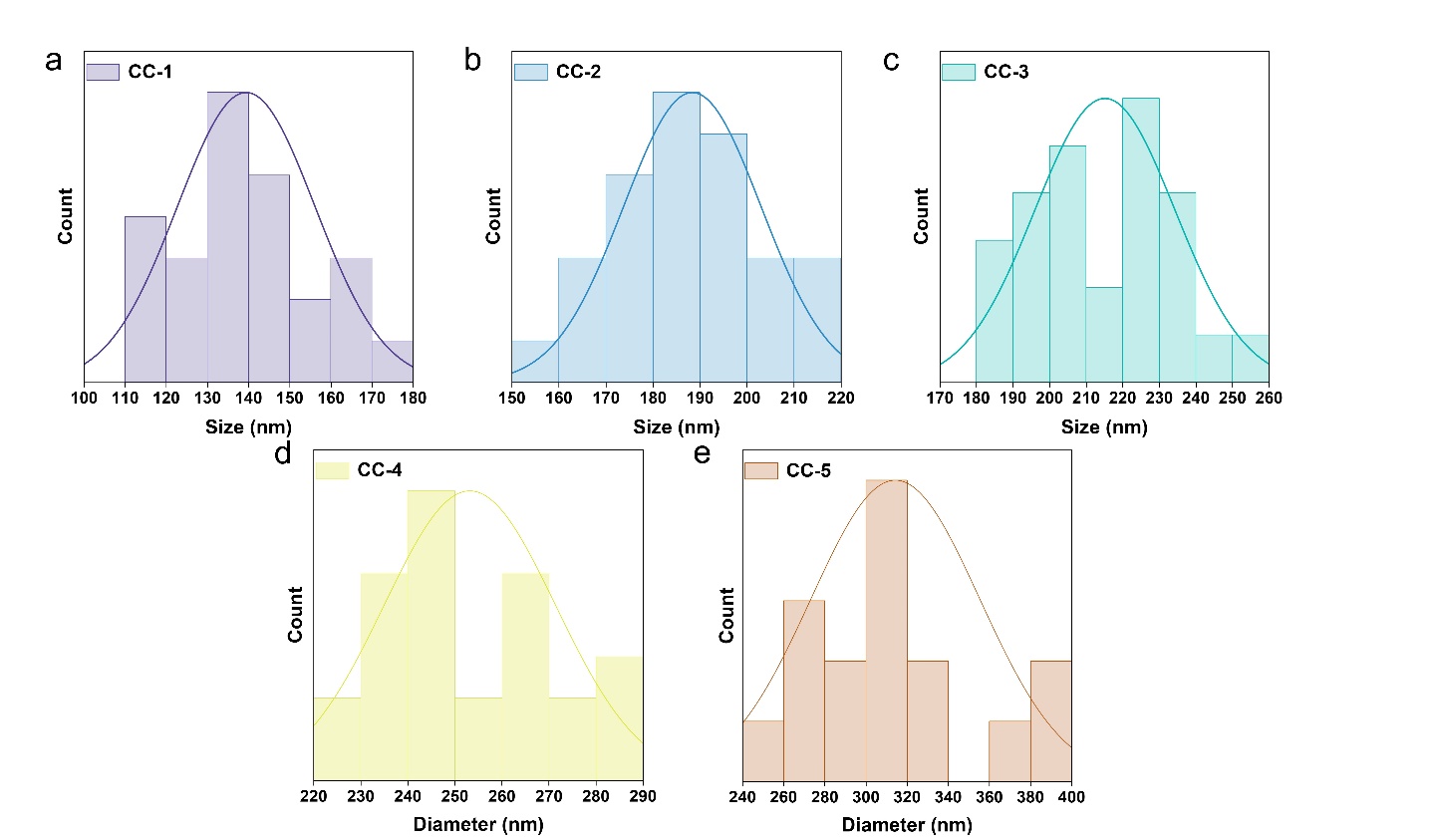


**Fig. S7** The diameter distribution of nanoparticles of **a** CC-1, **b** CC-2, **c** CC-3, **d** CC-4 and **e** CC-5


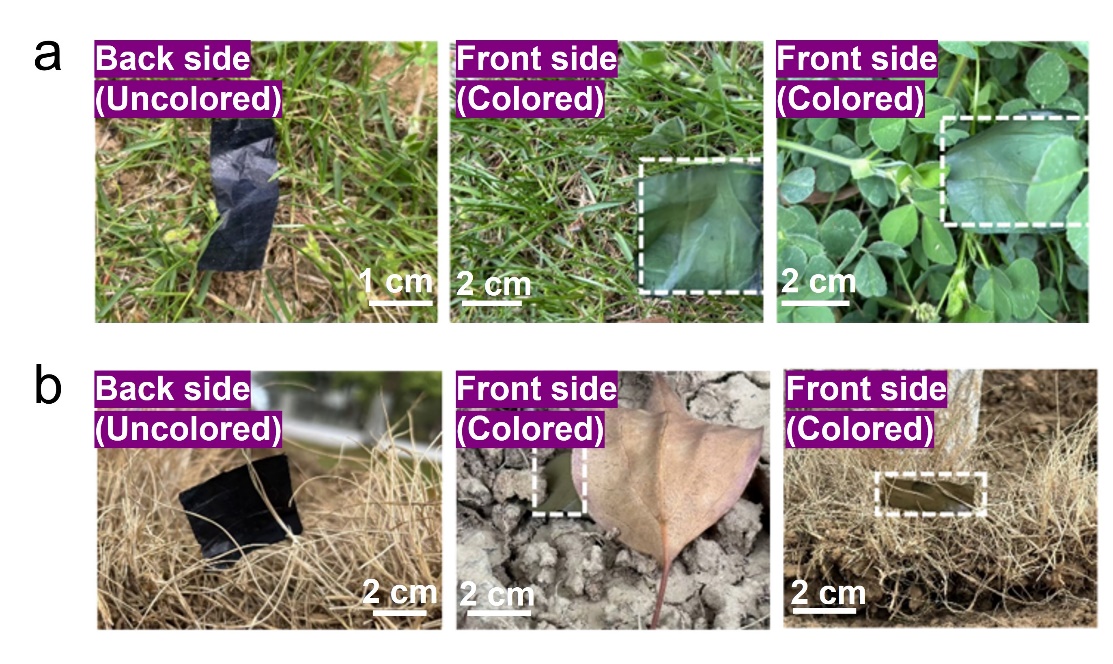


**Fig. S8** Scenario diagrams of visible camouflage for **a** CC-3 composite film and **b** CC-5 composite film

After six months of hermetic storage in sealed plastic bags, CC-1/2/3/4/5 composite films exposed to 85 °C and 85% relative humidity for one hour exhibit measurable spectral shifts in visible spectrum reflectance (Fig. S9). These changes include broadening of the reflection peak full width at half maximum (FWHM) and reduced overall reflectance, indicating partial fading of structural color. Crucially, characteristic reflection peaks persist at their original wavelengths, confirming the inherent environmental stability and long-term effectiveness of the structural coloration.


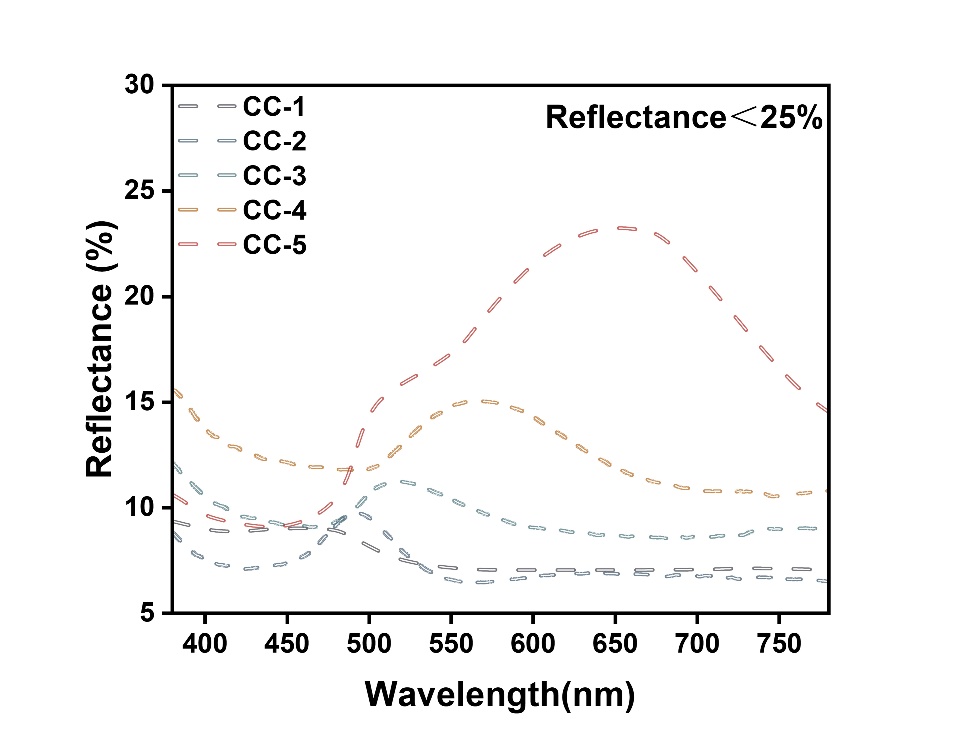


**Fig. S9** Reflectance spectrum of CC-1/2/3/4/5 composite films after exposed to 85 °C and 85% relative humidity for one hour

**
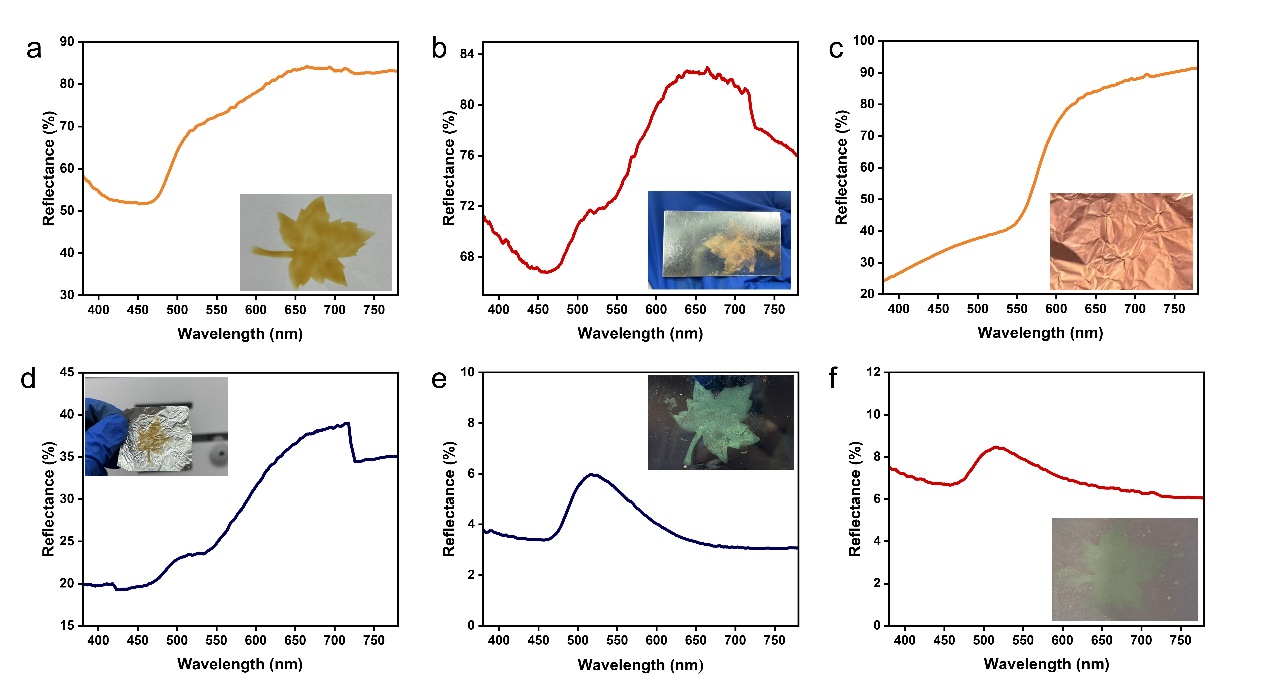
**

**Fig. S10** Digital photographs (spraying zone: maple leaf pattern) and reflectance spectra (380~780nm) of Cu_2_O nanoparticles (corresponding to CC-3) sprayed on **a** A4 paper substrate, **b** Al foil substrate, **c** Cu foil substrate, **d** Al sheet substrate, **e** pasteboard substrate and **f** cardboard substrate

To observe the layered structure of the device, a cross-sectional SEM test is conducted on the Cu_2_O/CNT/IL/CNT device (Fig. S11). During the cutting process before the test, fraying is inevitable because all three layers are flexible films, which leads to the unevenness of the layered structure in the image. Furthermore, due to the fact that the thickness of the nanoparticles can theoretically only reach approximately 1/300 of the device thickness, the surface nanoparticles cannot be observed.


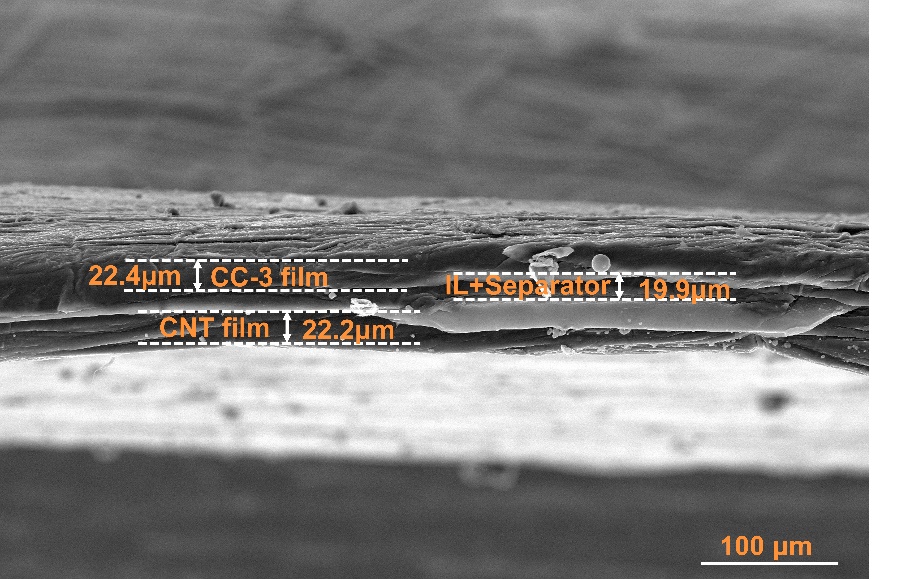


**Fig. S11** Cross-sectional SEM image of Cu_2_O/CNT/IL/CNT device (CC-3 as the representative)


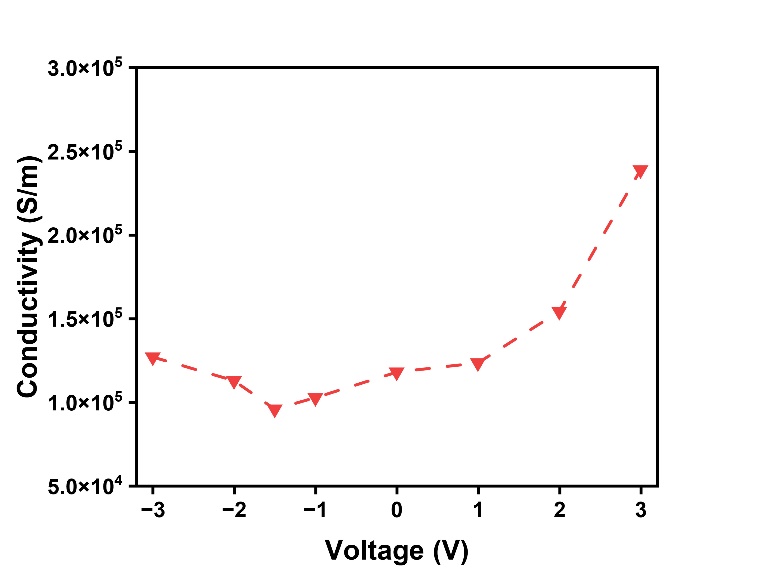


**Fig. S12** The variation of conductivity of the CC-3 composite film system under different voltages

Under hygrothermal (85°C Temperature/85% RH) conditions, the composite films display subtle modifications in infrared emissivity modulation performance, manifested by a reduction in maximum emissivity alongside an increase in minimum emissivity (Fig. S13a). Consequently, the modulation width experiences a marginal decrease while maintaining robust dynamic modulation capability. This behavior arises from competing mechanisms. Elevated temperature enhances charge carrier mobility, while adsorbed water molecules partially counteract this conductivity improvement, thereby suppressing maximum emissivity and elevating minimum emissivity.


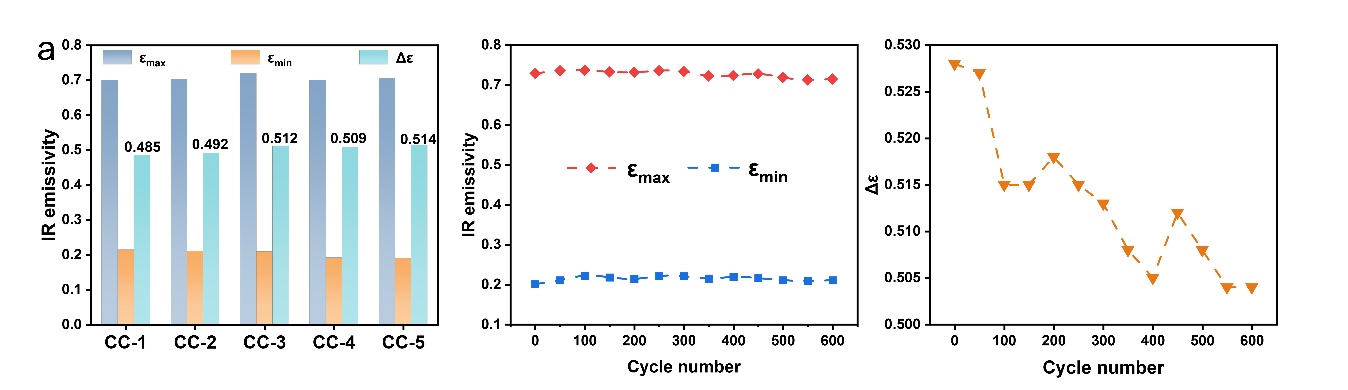


**Fig. S13** **a** IR modulation performance under humid and hot conditions. Cycle stability of IR electrochromic devices. **b** Maximum and minimum IR emissivity values. **c** Modulation width


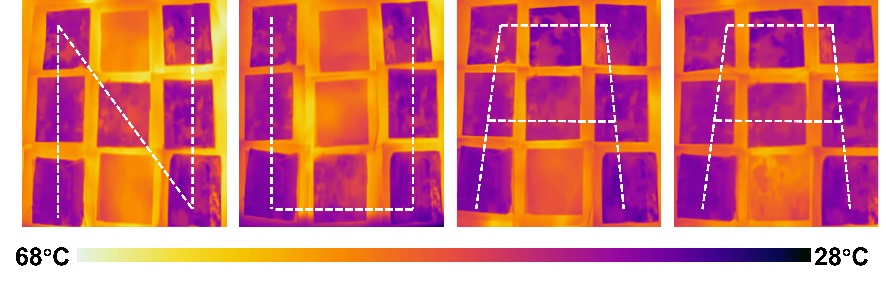


**Fig. S14** “Writing” characters "NUAA" (the abbreviation of Nanjing University of Aeronautics and Astronautics) in the LWIR band with array devices (array unit: 4×4 cm^2^)

Figure S15a, b respectively present the stress-strain curves of the composite film system under ambient conditions (25°C, 35% RH) and elevated temperature/humidity conditions (85°C, 85% RH), while Fig. S15 c,d quantitatively summarize the tensile strength, Young’s modulus, and elongation at break. At 25°C/35% RH, the system exhibits robust mechanical integrity with a tensile strength of 36 ± 1 MPa, maintains a Young’s modulus of ~0.4 GPa indicating uniform stiffness, and demonstrates good ductility through an average elongation at break of 9.5~10%. Under 85°C/85% RH, the tensile strength decreases to 31 ± 2 MPa, accompanied by a reduction in Young’s modulus to about 0.25 GPa, indicative of material softening and diminished rigidity. Concurrently, the average elongation at break increases to approximately 14.5%. This decrease in mechanical performance is attributed to weakened interfacial bonding between CNTs under hygrothermal conditions. Water vapor penetration likely disrupts van der Waals forces or interfacial interactions, while thermal activation promotes micro-defect expansion within the matrix. These synergistic effects facilitate CNTs slippage under stress, thereby extending the plastic deformation phase prior to fracture and manifesting enhanced elongation at break.


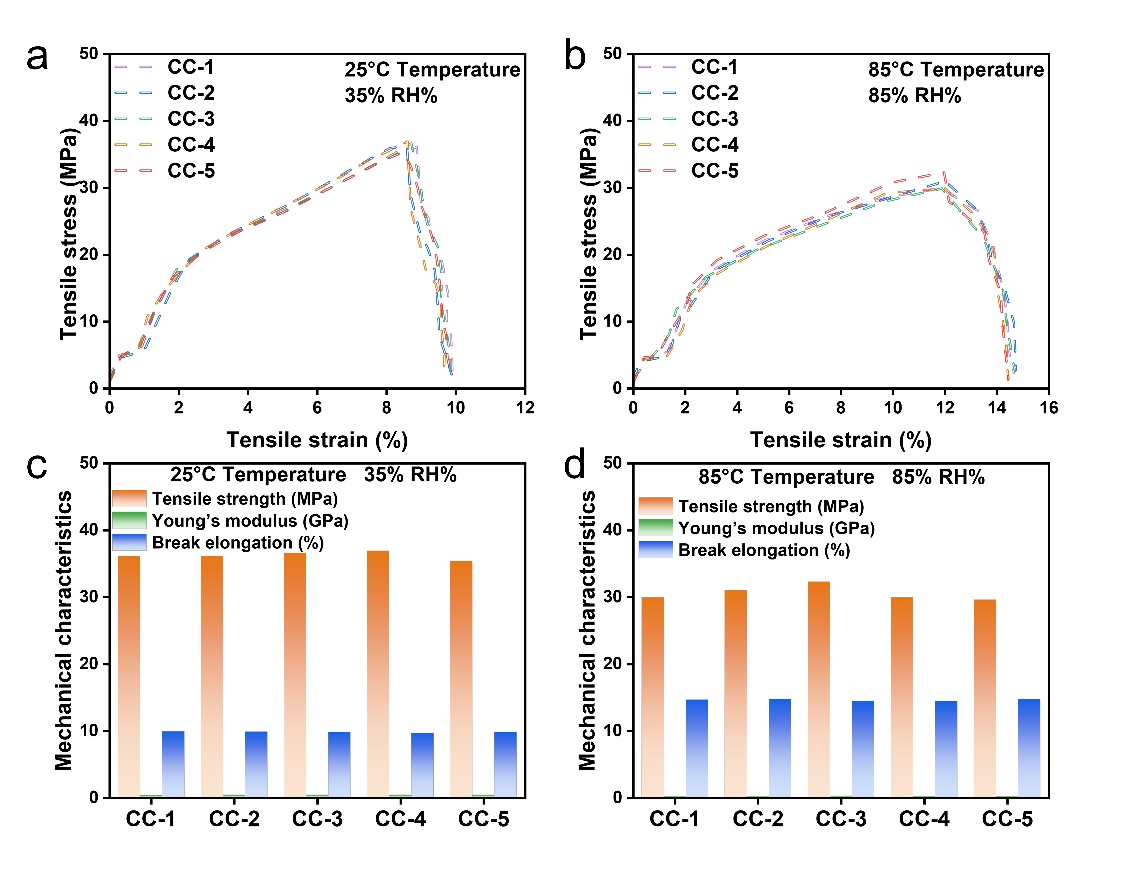


**Fig. S15 a** Stress-strain curves under the conditions of 25°C and 35% relative humidity. **b** Stress-strain curves under the conditions of 85°C and 85% relative humidity. **c** Tensile strength, Young's modulus and elongation at break under the conditions of 25°C and 35% relative humidity. **d** Tensile strength, Young's modulus and elongation at break under the conditions of 85°C and 85% relative humidity

Composite films follow the Joule heating effect in electrothermal conversion (Heating power $P=\frac{V^{2}}{R}$). When the voltage increases, the thermal power grows quadratically, significantly accelerating the initial heating rate ($\frac{ⅆT}{ⅆt}\propto\frac{V^{2}}{C}$)(C represents heat capacity). However, the time to reach the saturation temperature is prolonged instead due to the coupling effect between the heat dissipation kinetics and the material response. On the one hand, the system needs a larger temperature difference ΔT (ΔT = T_s_−T_0_) to balance enhanced heat source. Heat dissipation power ${(P}_{d}=hA\Delta T)$ approaches saturation in the high-temperature region, and T_s_ increases significantly with voltage increase ($T_{s}=T_{0}+\frac{V^{2}}{\mathrm{hAR}}$), resulting in a wider temperature range for thermal relaxation. On the other hand, the negative temperature coefficient of resistance of CNTs leads to reduced resistance at high temperatures, and the increase in power is partially offset by the increase in current. The time and space delay of thermal capacity hysteresis and local thermal diffusion further delay the establishment of overall thermal equilibrium. Eventually, this is manifested as a rapid initial temperature rise at high voltages but a prolonged process of approaching T_s_ [S10].


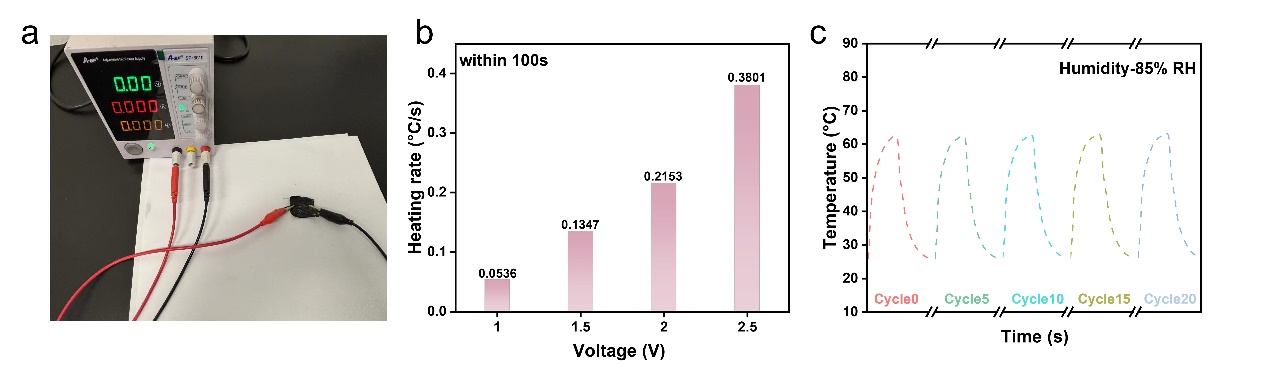


**Fig. S16** **a** Electrothermal conversion device. **b** E-heating rate within initial 100 s under different voltages (CC-3 as the representative). **c** Cyclic stability under high humidity conditions (CC-3 as the representative)


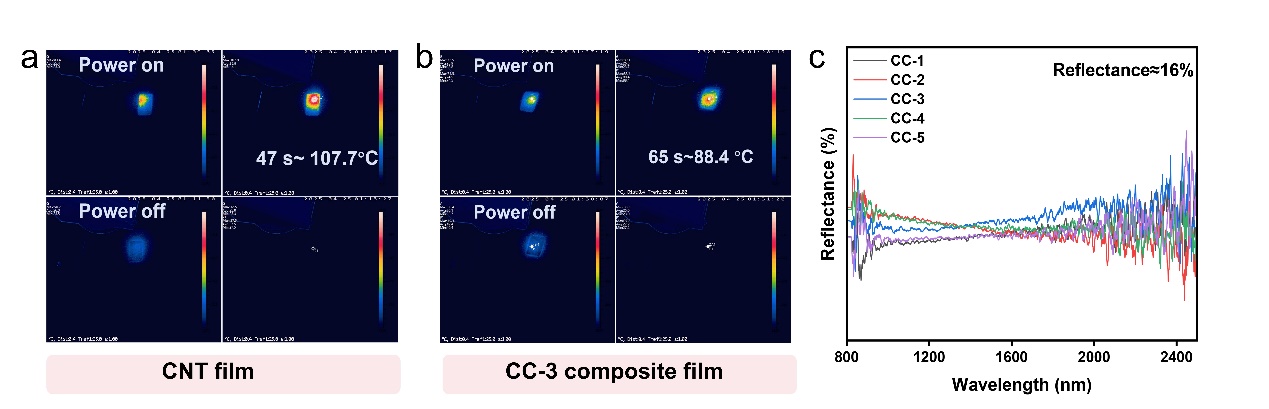


**Fig. S17** The IR temperature variation as response to light intensity of the **a** pristine CNT film and **b** CC-3 composite film. **c** Near-IR reflectance spectrum of the composite films


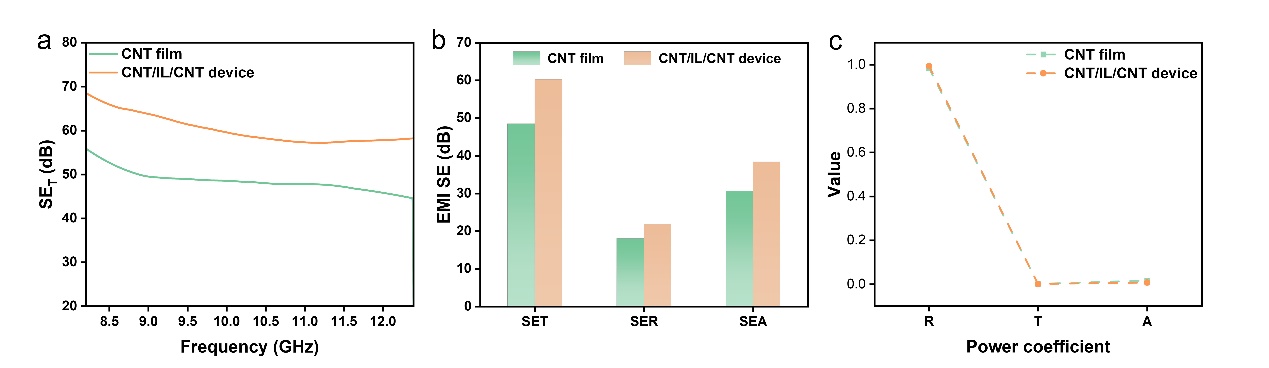


**Fig. S18** Pristine CNT film and CNT device **a** SE_T_ variation with frequency. **b** Average SE_T_, SE_R_, and SE_A_ values. **c** Power coefficients R, T and A

After placing the CC-n device in a high-temperature (85 °C) and high-humidity (85% RH) environment for 1 h, the EMI performance is tested (Fig. S19). Analysis of EMI shielding characteristics under these conditions reveals a slight decrease in total shielding effectiveness (SE_T_), primarily attributed to reduced reflection loss (SE_R_). This reduction in SE_R_ likely stems from water molecules partially penetrating the CNT network interstices, slightly increasing contact resistance. Meanwhile, absorption loss (SE_A_) remains stable or exhibits a slight increase, potentially due to transient dipole relaxation polarization induced by adsorbed water. Power coefficient analysis confirms that reflection loss driven by impedance mismatch continues to dominate the shielding mechanism under these hygrothermal conditions.


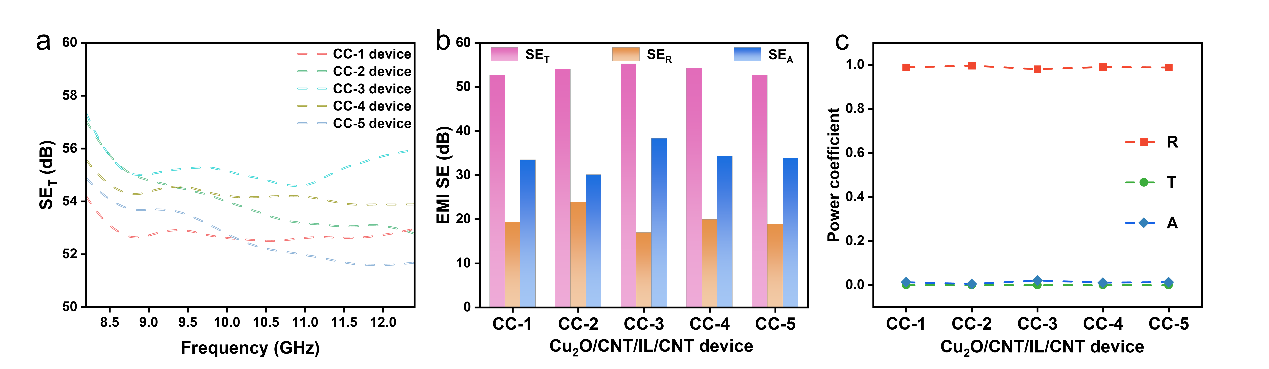


**Fig. S19** EMI shielding performance of CC-n device after placed in a high-temperature (85°C) and high-humidity (85% RH) environment. **a** The variation of the SE_T_ values with frequency. **b** Average SE_T,_ SE_R,_ SE_A_ values. **c** Power coefficients A, R, and T

Reflection spectrum shows that the functionalized CNT films (MACNT-1/2/3/4 films) still maintain a flat and low reflectivity, indicating that acidification has not altered their black characteristics (Fig. S20a). Based on the analysis of the coloring mechanism in **Section 2**, the structural coloring characteristics should not be affected. Cu_2_O nanoparticles (corresponding to the CC-3 formulation) were sprayed onto the functionalized films (MACC3-1/2/3/4 composite films), and consistent reflection peaks were observed at around 520 nm, further demonstrating the uncompromised color presentation (Fig. S20b). After assembling the MACC3-1/2/3/4 composite films into devices, the modulation width remained stably greater than 0.47 (Fig. S20c). Furthermore, the functionalized composite film exhibits even more superior electrothermal conversion performance (Fig. S20d-e), which is attributed to the enhanced electrical conductivity (Fig. S20f).

The electrical conductivity of functionalized CNT films increases from 985 S/cm (MACNT-1) to 1163 S/cm (MACNT-3) as the concentration of acid increases, and then decreases to 1068 S/cm (MACNT-4), which consistently exceed that of pristine CNT film (959 S/cm) (Fig. S20f). This non-monotonic behavior arises from competing interfacial phenomena. Moderate introduction of oxygen functional groups (-OH, -COOH) promotes hydrogen-bond-mediated inter-tube bridging, reducing contact resistance through improved electron tunneling efficiency and physical packing densification. Beyond optimal functionalization, excessive surface oxidation partially disrupts the sp^2^-conjugated lattice, diminishing electrical conductivity.


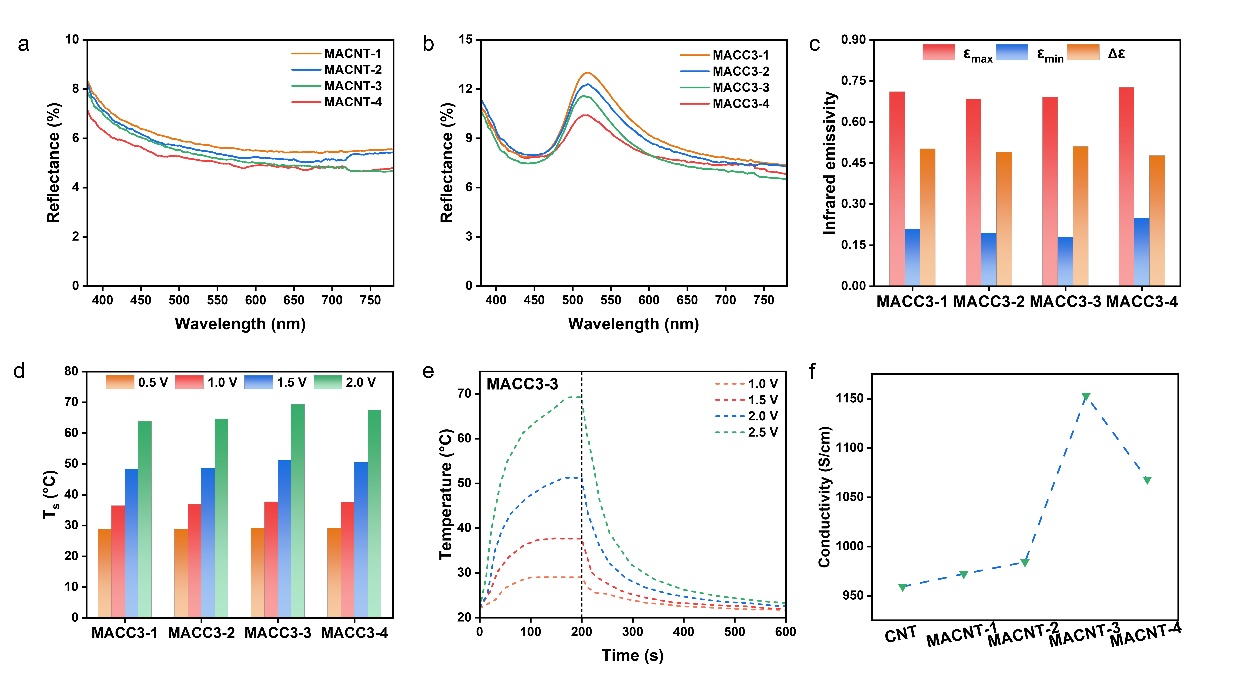


**Fig. S20 a** Reflection spectra (380~780 nm) of MACNT-1/2/3/4 films. **b** Reflection spectra (380~780 nm) of MACC3-1/2/3/4 composite films. **c** IR electrochromic properties of MACC3-1/2/3/4 composite films. **d** Saturation temperature under different voltages of MACC3-1/2/3/4 composite films. **e** Electrothermal conversion curves of MACC3-1/2/3/4 composite films. **f** Conductivity of pristine and functionalized CNT films

**
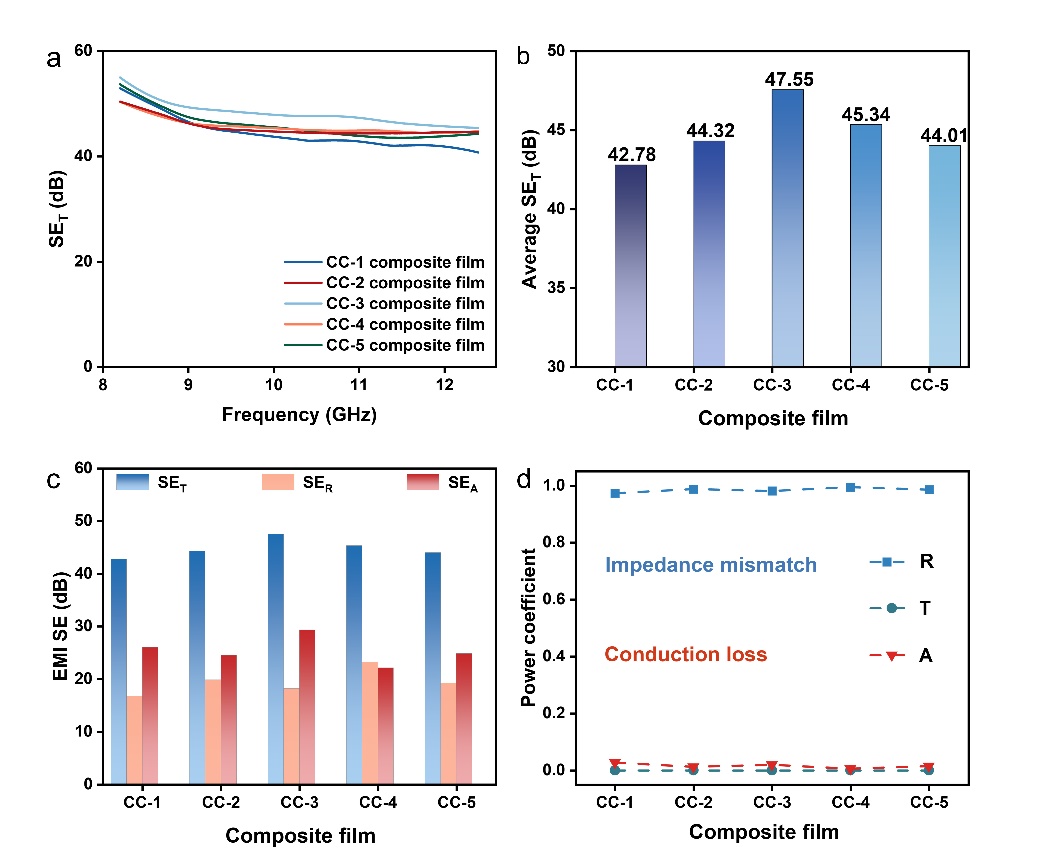
**

**Fig. S21** CC-1/2/3/4/5 composite films **a** The variation of the SE_T_ values with frequency. **b** Average SE values. **c** Average SE_T,_ SE_R,_ SE_A_ values. **d** Power coefficients A, R, and T

**
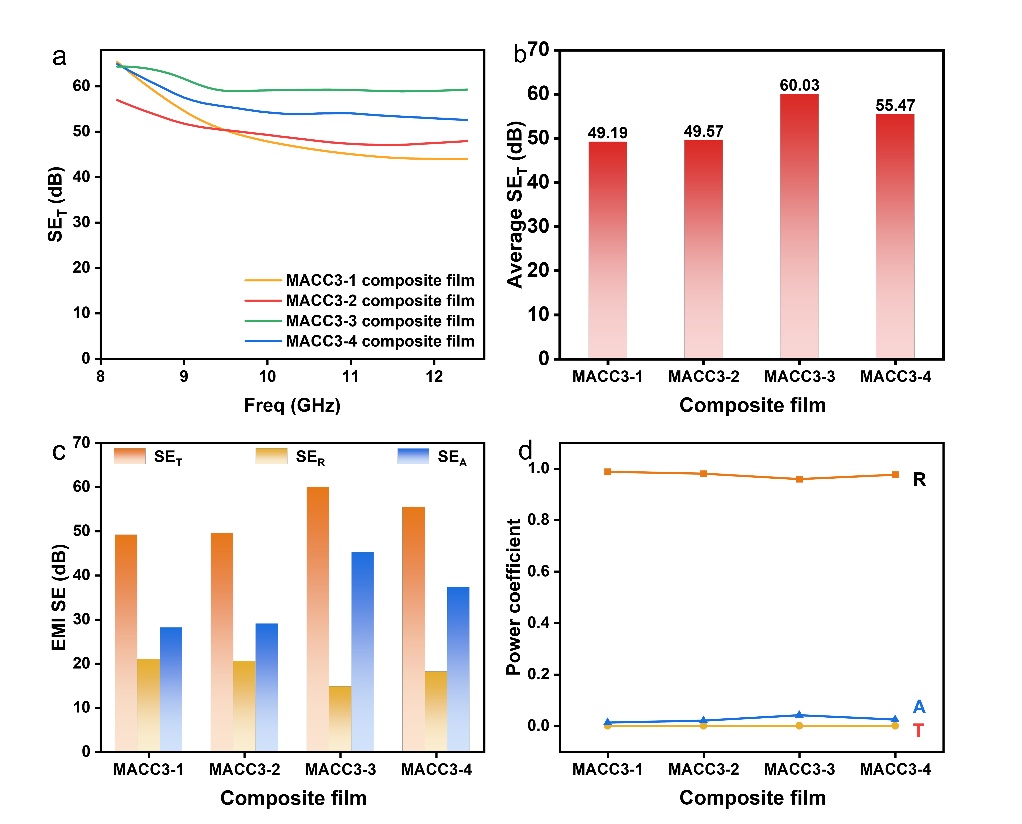
**

**Fig. S22** MACC3-1/2/3/4 composite films **a** The variation of the SE_T_ values with frequency. **b** Average SE values. **c** Average SE_T,_ SE_R,_ SE_A_ values. **d** Power coefficients A, R, and T

**
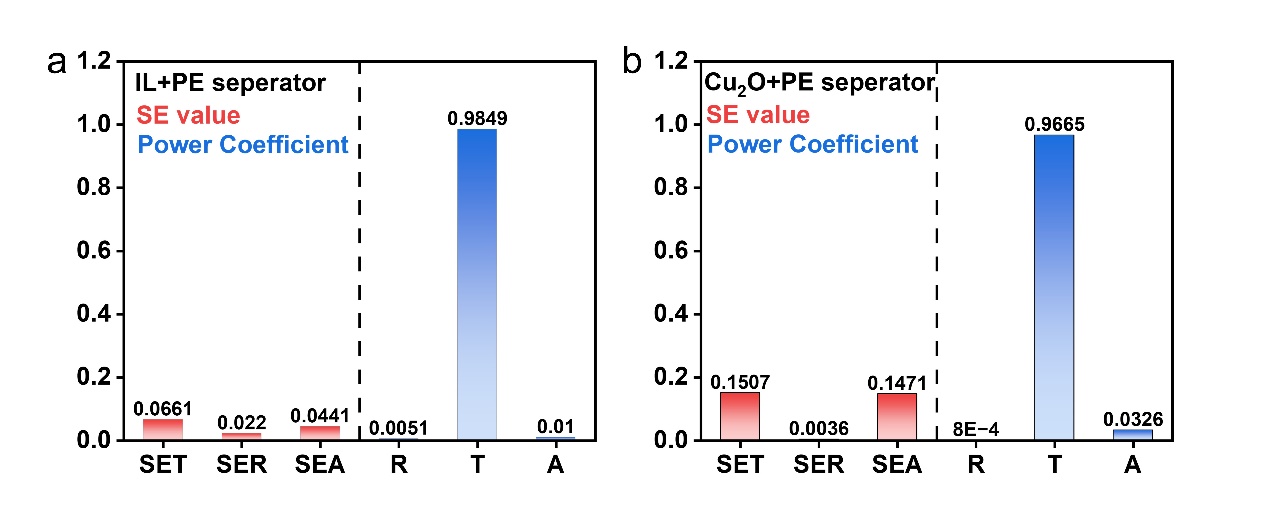
**

**Fig. S23 a** EMI SE values and power coefficient of IL-immersed separator. **b** EMI SE values and power coefficient of Cu_2_O-coated separator

After spraying different numbers of layers (5, 10, 15, and 30 layers) of Cu_2_O nanoparticles (CC-3 formulation) on the surface of the CNT film, we conduct SEM tests (Fig. S24). The thickness of the surface nanoparticles do not increase with the increase in the number of spraying layers, but the packing density is constantly rising. Fig. S25 a-c presents the EMI shielding performance of four composite films with different spraying densities. As the packing density of the nanoparticles increases, the SE_T_ value decreases slightly, mainly due to the decline in SE_A_.

Above phenomenon can be explained by the theory of effective medium (EMT). Herein, we consider the composite system of "CNT film + Cu_2_O nanoparticles" as an equivalent macroscopic uniform "effective medium", whose electromagnetic parameters (such as conductivity, dielectric constant) are determined jointly by the volume fractions of the two phases (carbon nanotube phase and cuprous oxide phase) and their own electromagnetic properties.

CNTs are the high-conductivity phase (dominating the absorption loss of electromagnetic waves), while Cu_2_O is the low-conductivity semiconductor phase (contributing almost no absorption). When the amount of Cu_2_O nanoparticles increases, it is equivalent to introducing a large number of "low-conductivity impurities" into the continuous conductive network of carbon nanotubes, which disrupts the continuous conductive pathways between carbon nanotubes and leads to a significant decrease in the equivalent conductivity of the composite system. And SE_A_ is positively correlated with the conductivity (the lower the conductivity, the less Joule heat dissipation of electromagnetic waves within the material), so SE_A_ decreases with the increase in particles (since the nanoparticles are only nano-level stacking, the degree of damage to the network is limited, so SE_A_ only decreases minimally).

The thickness of the Cu_2_O nanoparticle layer is at the nanometer scale, much smaller than the 20 μm of the CNT film, and its volume fraction is extremely low. The macroscopic surface impedance of the composite system is mainly determined by the dominant CNT film (the 20-μm-thick continuous phase), and the low volume fraction of Cu_2_O cannot change the overall equivalent impedance characteristics, so the surface impedance is not affected and SE_R_ remains basically unchanged.


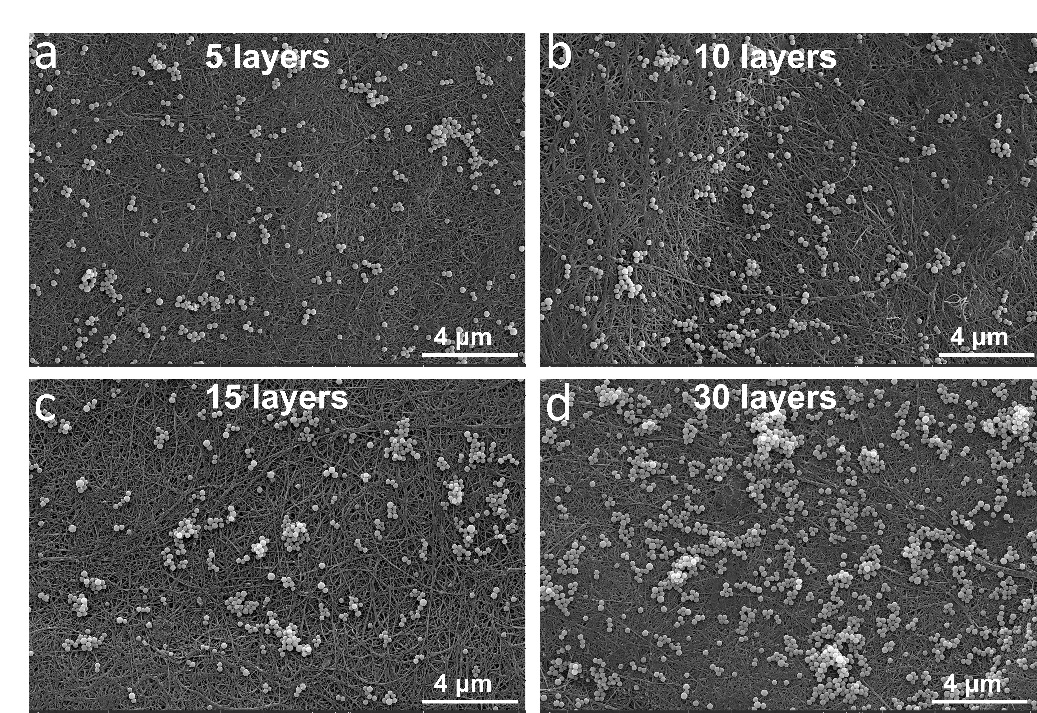


**Fig. S24** SEM images of CNT film coated with different layers of Cu_2_O (CC-3 formula) nanoparticles. **a** 5 layers. **b** 10 layers. **c** 15 layers. **d** 30 layers


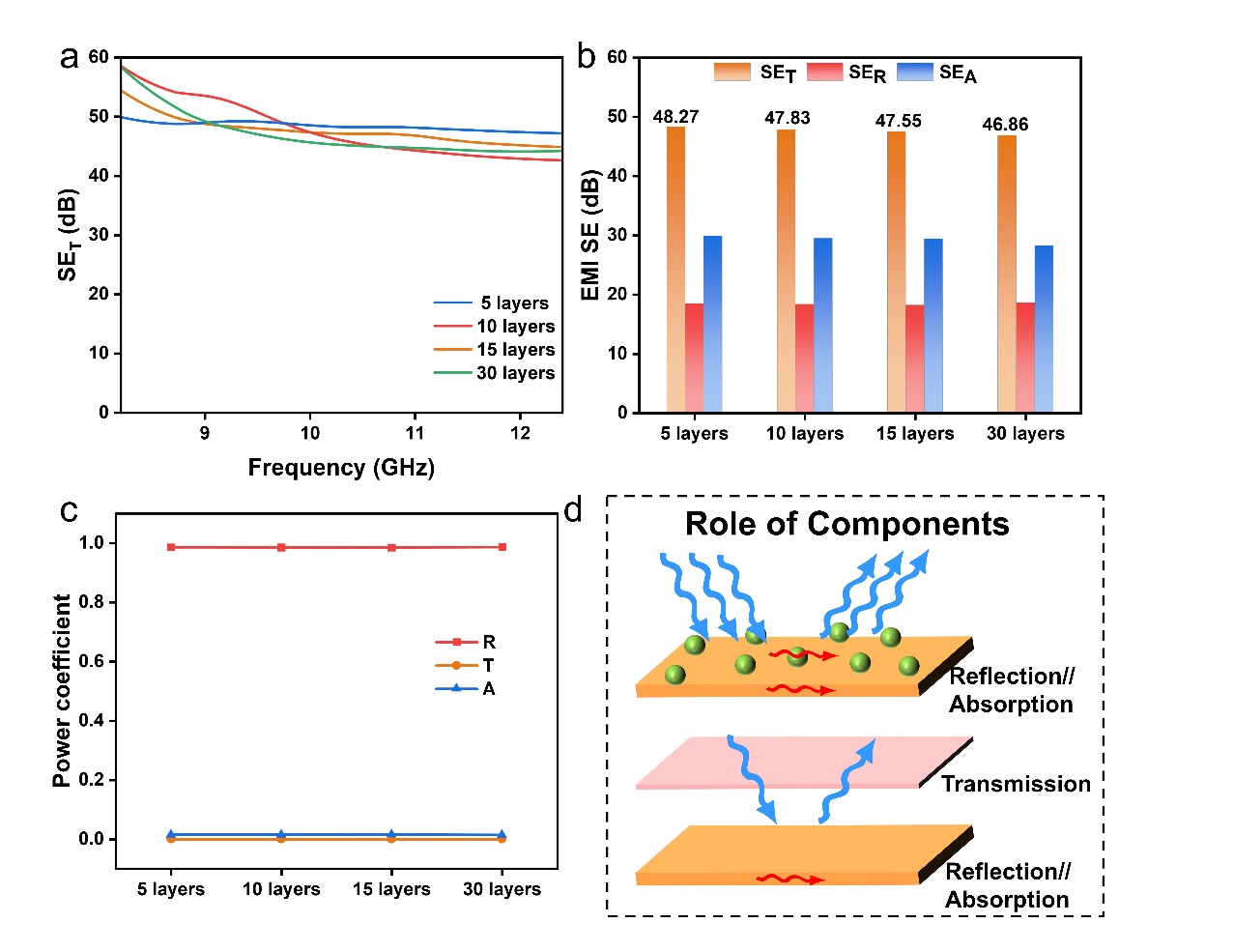


**Fig. S25** EMI shielding performance of CNT film coated with different layers of Cu_2_O (CC-3 formula) nanoparticles. **a** The variation of the SE_T_ values with frequency. **b** Average SE_T,_ SE_R,_ SE_A_ values. **c** Power coefficients A, R, and T. **d** Contribution of each component in the device to the EMI shielding performance


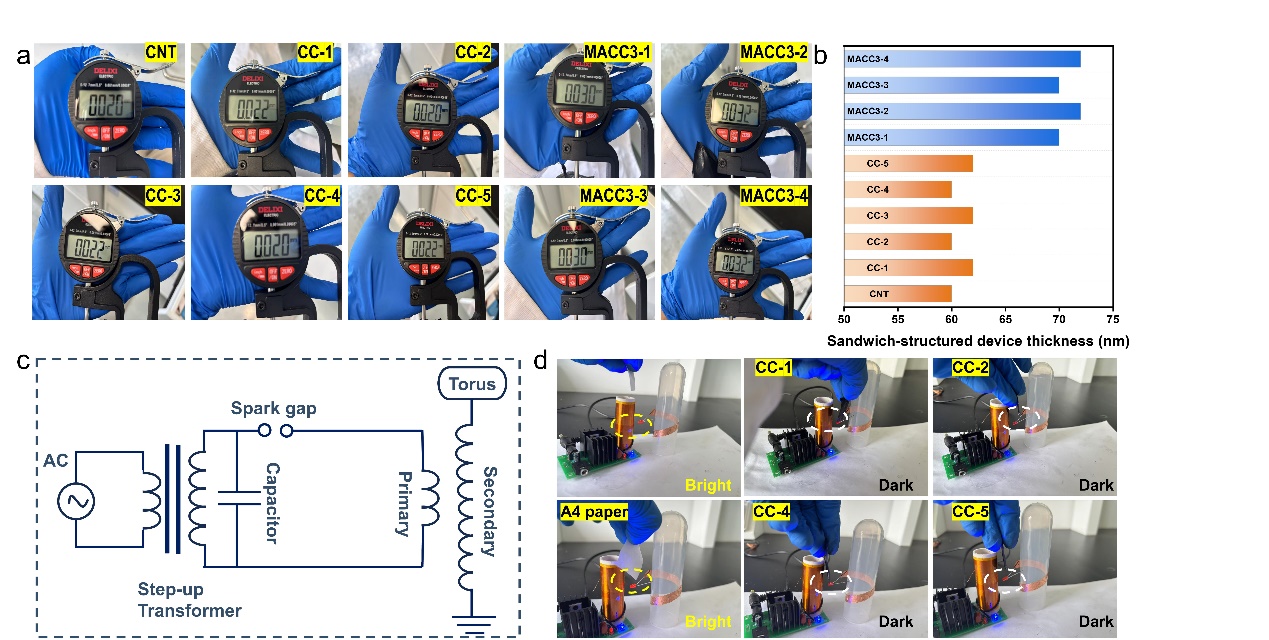


**Fig. S26** Thickness of **a** Composite films and **b** CC-n devices and MACC3-n devices. Wireless power transmission circuit **c** The working principle diagram of the Tesla coil. **d** Comparison of bulb conditions when not inserted, when inserted into A4 paper, and when inserted into the CC films system


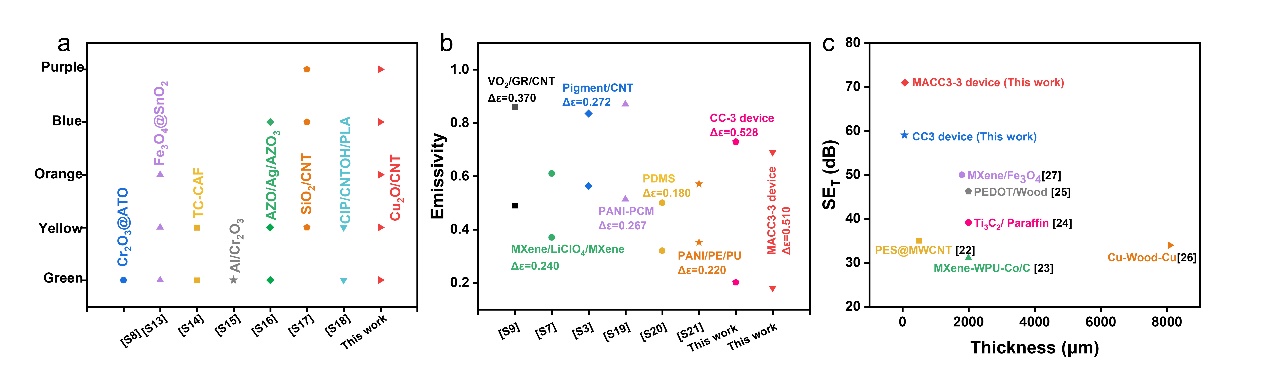


**Fig. S27** Comparation with other military defense systems regarding **a** rich coloration. **b** IR modulation width. **c** EMI interference shielding effectiveness

As shown in Fig. S28, we investigate the multispectral synchronous integration characteristics of the proposed design system. The airplane is against a background of plants, and due to the difference in color, it becomes visually conspicuous. After covering the CC-3 device, the covered area blends in with the dark green plant background. Since the infrared radiation signal of the aircraft is more prominent, we apply a +2V voltage to the CC-3 device. The reduction in its infrared emissivity causes the infrared radiation signal of the covered area to match that of the background. Furthermore, during the voltage application process, due to the increase in conductivity, the EMI shielding performance reaches 78 dB, effectively blocking external electromagnetic interference and internal important information leakage. Therefore, this device achieves an uncompromisingly integrated multi-functionality.


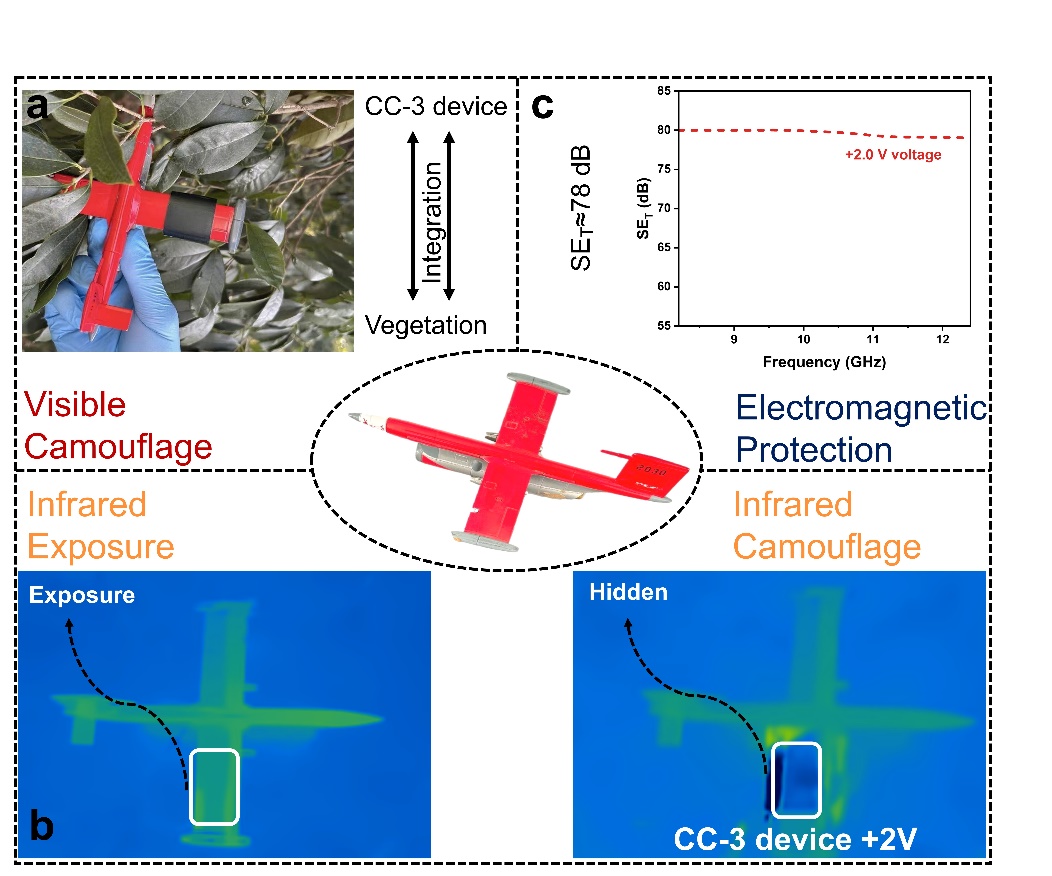


**Fig. S28** The multispectral integration characteristics of the CC-3 device in the vegetation background. **a** Visible camouflage. **b** IR camouflage. **c** EMI interference shielding

To further enhance color stability, 5 wt % polyurethane (PU) and polyvinyl alcohol (PVA) aqueous solutions are both pre-deposited onto CNT films prior to Cu_2_O dispersion spraying (corresponding to CC-3), yielding CC3Un and CC3Pn composite films (where n denotes corresponding polymer deposition layer number), respectively. Both systems exhibit parallel behavior that polymer pre-treatment maintains original reflectance peak positions while progressively reducing reflectance with increasing deposition layers (Fig. S29 a,b). Thermal characterization at 60°C reveals IR radiation temperatures of 58.8°C (CC-3), 59.2°C (CC3P7), and 60.4°C (CC3U7) (Fig. S29 c). The relatively tiny IR radiation deviation in PVA-modified composite film indicates better IR transmittance. Therefore, PVA is chosen as the material for enhancing stability. PVA-modified CC3P7 composite film demonstrates exceptional color retention following friction testing (50g load, 20cm stroke), while CC4P7 (PVA aqueous solution is pre-deposited onto CNT film prior to spraying of Cu_2_O dispersion corresponding to CC-4) also maintains structure color integrity through 50 bending cycles despite visual surface creasing (Fig. S30a). SEM image confirms continuous PVA-CNTs interfacial coupling with complete encapsulation of nanotube bundles (Fig. S30b). Crucially, IR emissivity modulation remains effective (Δε = 0.466 for CC3P7, Δε = 0.469 for CC4P7, Fig. S30c). To enhance its tolerance to humid environments, a hydrophobic coating is fabricated by ultrasonically dissolving 5 g PDMS in 95 g isopropanol, followed by sequential spray deposition of seven layers onto the surface of CC3P7 film, thereby achieving a water contact angle exceeding 115°, further enhancing application extensibility (Fig. S30d). Furthermore, we compared the visible light reflection properties of the CC-3 composite film and the CC3P7 composite film coated with PDMS. It can be seen that the polymer modification does not affect the effective presentation of structure color (Fig. S30e). Uniform structure coloration achieves across 56 cm × 52 cm black textile substrates via spray deposition (0.2425g Cu_2_O, properly reduce the concentration of the dispersion solution), validating industrial-scale process compatibility (Fig. S30f).


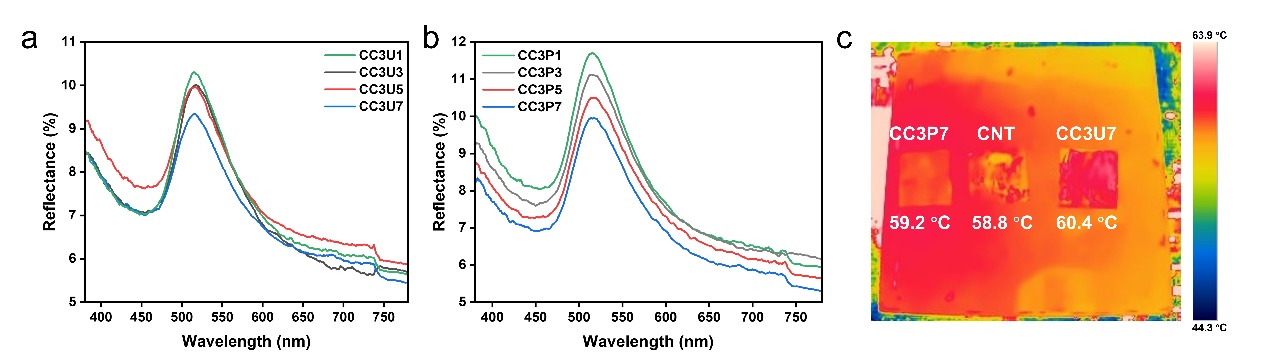


**Fig. S29** Reflectance spectra (380~780 nm) of CC-3 composite films with different numbers of pre-sprayed a PU layers, and b PVA layers. c IR thermal images comparison of CC-3, CC3U7, and CC3P7 composite films


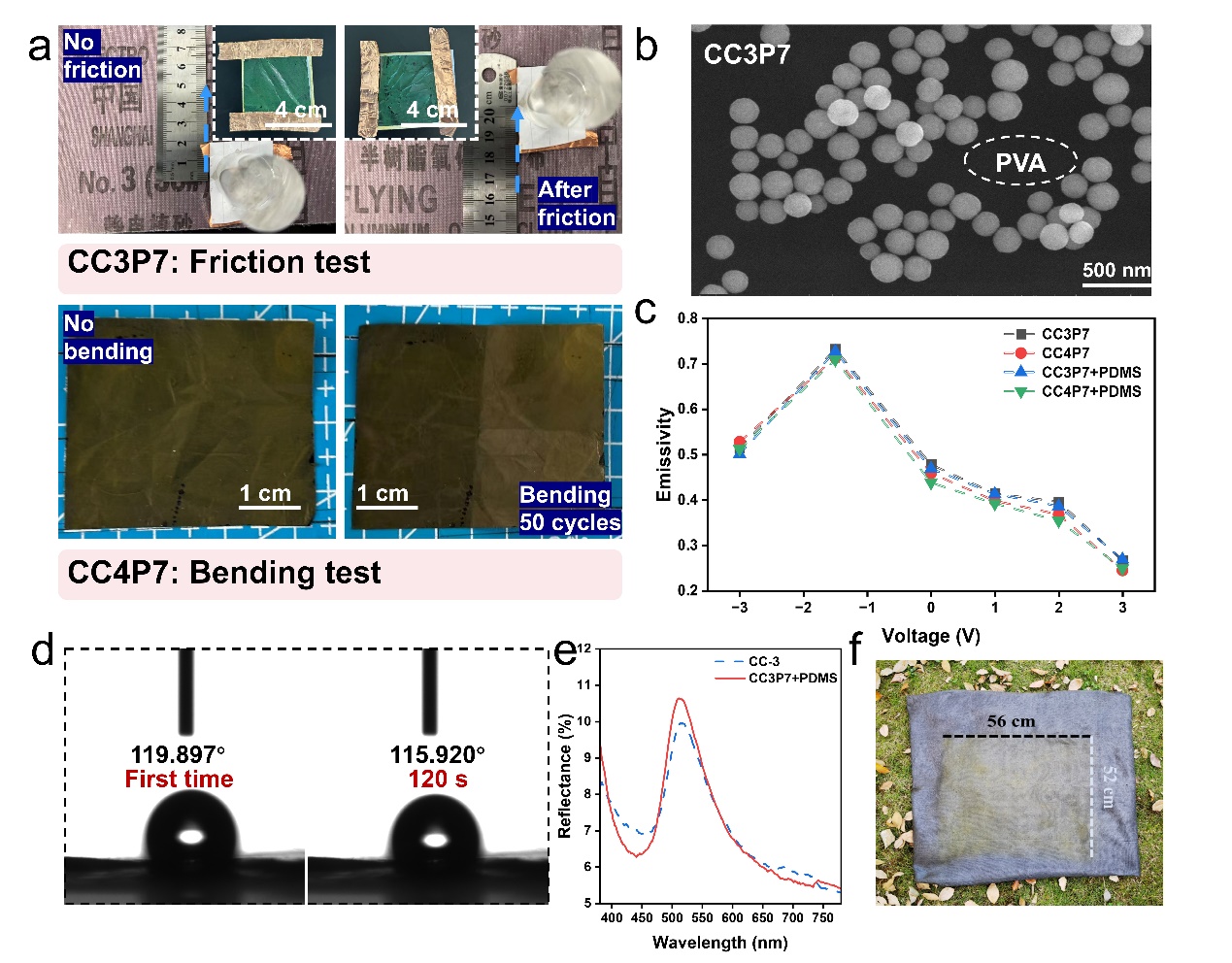


**Fig. S30 a** Friction test of CC3P7 and bending test of CC4P7 composite films. **b** SEM image of CC3P7 composite film. **c** Infrared emissivity changes of the CC3P7 and CC4P7 composite films with the applied voltage before and after PDMS coating. **d** The hydrophobic property of the PDMS-modified CC3P7 composite film. **e** Comparison of reflection spectra (380~780 nm) of CC3P7 before and after coating with PDMS. **f** Scale-up coloration (based on fabric substrate).

**Supplementary References**

1. X. Yu, G. Bakan, H. Guo, M. Ergoktas, P. Steiner et al., Reversible ionic liquid intercalation for electrically controlled thermal radiation from graphene devices. ACS Nano **17**, 11583-11592 (2023). <https://doi.org/10.1021/acsnano.3c01698>
2. R. Pankow, A. Harbuzaru, D. Zheng, B. Kerwin, G. Forti, et al., Oxidative-reductive near-infrared electrochromic switching enabled by porous vertically stacked multilayer devices. J. Am. Chem. Soc. **145**, 13411-13419 (2023). <https://doi.org/10.1021/jacs.3c03702>
3. G. Feng, H. Chang, S. Tan, L. Qu, T. Chen et al., Colorization strategy for carbon-based electrochromic devices: Achieving visible and mid-infrared regions modulation. Carbon **224**, 119084, (2024). <https://doi.org/10.1016/j.carbon.2024.119084>
4. Y. Zhang, Y. Feng, J. Li, T. Xu, Y. Wu et al., Multi-interfacial bridging engineering of flexible mxene film for efficient electromagnetic shielding and energy conversion. J. Colloid Interf. Sci. **665**, 733-741 (2024). <https://doi.org/10.1016/j.jcis.2024.03.173>
5. Y. Xiong, Y. Zhou, J. Tian, W. Wang, W. Zhang et al., Scalable, color‐matched, flexible plasmonic film for visible–infrared compatible camouflage. Adv. Sci. **10**, 2303452 (2023). <https://doi.org/10.1002/advs.202303452>
6. H. Peng, S. Tan, Y. Zhao, X. Guan, M. Li et al., A multi-scale hierarchically omnidirectional strategy to elevate crossband camouflage and anti-icing properties. Chem. Eng. J. **500**, 157515 (2024). <https://doi.org/10.1016/j.cej.2024.157515>
7. F. Lu, D. Shi, P. Tan, Y. Han, A novel infrared electrochromic device based on ti3c2t mxene. Chem. Eng. J. **450**, 138324 (2022). <https://doi.org/10.1016/j.cej.2022.138324>
8. X. Chai, D. Zhu, Y. Liu, Y. Qing, F. Luo et al., In-situ construction of Cr_2_O_3_@ATO hybrid pigment towards synergetic enhancement of visible light-infrared-radar compatible stealth. J. Colloid Interf. Sci. **645**, 570-579 (2023). <https://doi.org/10.1016/j.jcis.2023.04.175>
9. L. Xiao, H. Ma, J. Liu, W. Zhao, Y. Jia et al., Fast adaptive thermal camouflage based on flexible VO_2_/graphene/cnt thin films. Nano Lett. **15**, 8365-8370 (2015). <https://doi.org/10.1021/acs.nanolett.5b04090>
10. X. Gao, J. Wang, X. Li, Comparative research on two surface conductivity models for the scattering of electromagnetic wave by the charged sphere. J. Quant. Spectrosc Ra. **224**, 378-382 (2019). <https://doi.org/10.1016/j.jqsrt.2018.11.040>
11. C. Zhang, Y. Liu, Electron-surface scattering from first-principles. ACS Nano **18**, 27433-27439 (2024). <https://doi.org/10.1021/acsnano.4c07698>
12. C. Karaman, A. Bykov, F. Kiani, G. Tagliabue, A. Zayats, Ultrafast hot-carrier dynamics in ultrathin monocrystalline gold. Nat. Commun. **15**, 703 (2024). <https://doi.org/10.1038/s41467-024-44769-3>
13. M. Qiao, Y. Tian, J. Li, X. He, X. Lei, et al., Core-shell Fe(3)O(4)@SnO(2) nanochains toward the application of radar-infrared-visible compatible stealth. J. Colloid Interf. Sci. **609**, 330-340 (2022). <https://doi.org/10.1016/j.jcis.2021.12.012>
14. Y. Pan, X. Hu, C. Ye, M. Zhu, Bilayer smart and multifunctional camouflage textiles integrating adaptive visible stealth, infrared concealment, and electromagnetic interference shielding. ACS Appl. Polym. Mater. **7**, 7350-7359 (2025). <https://doi.org/10.1021/acsapm.5c00910>
15. X. Chai, D. Zhu, Q. Chen, Y. Qing, K. Cao, et al. Tailored composition of low emissivity top layer for lightweight visible light-infrared-radar multiband compatible stealth coating. [Adv. Compos. Hybrid Ma.](https://link.springer.com/journal/42114) **5**, 3094–3103, (2022). http://doi.org/ 10.1007/s42114-022-00563-7
16. L. Wang, L. Hu, W. Wang, G. Liu, et al., Electrochromic and thermal-control broadband stealth device based on azo/ag/azo configuration electrodes. Chinese J. Phys. **89**, 1890-1898 (2024). <https://doi.org/10.1016/j.cjph.2024.05.016>
17. J. Liu, G. Feng, S. Tan, M. Zhou, T. Chen et al., Dual-band dynamically compatible stealth: Infrared modulation and visible camouflage based on carbon nanotubes. J. Alloys. Compd. **1020**, 179525, (2025). <https://doi.org/10.1016/j.jallcom.2025.179525>
18. S. Guo, S. Tan, X. Zhang, X. Huang, G. Ji, Pyramid-like magnetic carbon composites device toward tunable and adaptive radar-visible compatible properties. Carbon **231**, (2025). <https://doi.org/10.1016/j.carbon.2024.119737>
19. Z. Zhang, L. Zhang, Z. Ren, Y. Zhang, T. Hao, et al., Multifunctional ultrathin metasurface with a low radar cross section and variable infrared emissivity. ACS Appl. Mater. Interfaces **16**, 21109-21117 (2024). <https://doi.org/10.1021/acsami.4c01798>
20. Z. Li, L. Long, Z. Tang, X. Chen, Z. Huang, et al., Stretchable metamaterials with tunable infrared emissivity for dynamic thermal management. ACS Appl. Mater. Interfaces **16**, 47639-47645 (2024). <https://doi.org/10.1021/acsami.4c09758>
21. J. Wu, X. Tang, H. Xu, Y. Zhong, L. Zhang, et al., Temperature responsiveness-hygroscopic infrared stealth composite film with variable emissivity. Appl. Mater. Today **45**, (2025). <https://doi.org/10.1016/j.apmt.2025.102814>
22. N. Abbas, H. Kim, Multi-walled carbon nanotube/polyethersulfone nanocomposites for enhanced electrical conductivity, dielectric properties and efficient electromagnetic interference shielding at low thickness. Macromol. Res. **24**, 1084-1090 (2016). <https://doi.org/10.1007/s13233-016-4152-z>
23. G. Zhang, M. Du, Z. Tan, H. Zhao, L. Yi, et al., Structure design and properties of mxene-wpu-co/c electromagnetic shielding composite films. Mater. Today Commun. **37**, (2023). <https://doi.org/10.1016/j.mtcomm.2023.107536>
24. X. Liu, J. Wu, J. He, L. Zhang, Electromagnetic interference shielding effectiveness of titanium carbide sheets. Mater. Lett. **205**, 261-263 (2017). <https://doi.org/10.1016/j.matlet.2017.06.101>
25. C. Chen, W. Feng, W. Wu, Y. Yu, G. Qian, et al., A highly strong pedot modified wood towards efficient electromagnetic interference shielding. Ind. Crop. Prod. **202**, (2023). <https://doi.org/10.1016/j.indcrop.2023.117109>
26. A. Wei, Y. Ding, C. Hu, C. Wang, X. Lin, et al., Enhancing the performance of high-efficiency electromagnetic shielding via controlling the thickness of balsa wood in the fp cavity structure. J. Mater. Sci-Mater. EL. **36**, (2025). <https://doi.org/10.1007/s10854-025-14731-0>
27. H. Zhang, J. Cheng, K. Liu, S. X. Jiang, J. Zhang, et al., Electric-magnetic dual-gradient structure design of thin mxene/Fe_3_O_4_ films for absorption-dominated electromagnetic interference shielding. J. Colloid Interf. Sci. **678**, 950-958 (2025). <https://doi.org/10.1016/j.jcis.2024.08.216>
